# The ORC/Cdc6/MCM2-7 complex facilitates MCM2-7 dimerization during prereplicative complex formation

**DOI:** 10.1093/nar/gkt1148

**Published:** 2013-11-14

**Authors:** Cecile Evrin, Alejandra Fernández-Cid, Alberto Riera, Juergen Zech, Pippa Clarke, M. Carmen Herrera, Silvia Tognetti, Rudi Lurz, Christian Speck

**Affiliations:** ^1^DNA Replication Group, MRC Clinical Sciences Centre, Imperial College, Du Cane Road, London W12 0NN, UK and ^2^Microscopy Unit, Max Planck Institute for Molecular Genetics, Berlin 14195, Germany

## Abstract

The replicative mini-chromosome-maintenance 2–7 (MCM2-7) helicase is loaded in *Saccharomyces cerevisiae* and other eukaryotes as a head-to-head double-hexamer around origin DNA. At first, ORC/Cdc6 recruits with the help of Cdt1 a single MCM2-7 hexamer to form an ‘initial’ ORC/Cdc6/Cdt1/MCM2-7 complex. Then, on ATP hydrolysis and Cdt1 release, the ‘initial’ complex is transformed into an ORC/Cdc6/MCM2-7 (OCM) complex. However, it remains unclear how the OCM is subsequently converted into a MCM2-7 double-hexamer. Through analysis of MCM2-7 hexamer-interface mutants we discovered a complex competent for MCM2-7 dimerization. We demonstrate that these MCM2-7 mutants arrest during prereplicative complex (pre-RC) assembly after OCM formation, but before MCM2-7 double-hexamer assembly. Remarkably, only the OCM complex, but not the ‘initial’ ORC/Cdc6/Cdt1/MCM2-7 complex, is competent for MCM2-7 dimerization. The MCM2-7 dimer, in contrast to the MCM2-7 double-hexamer, interacts with ORC/Cdc6 and is salt-sensitive, classifying the arrested complex as a helicase-loading intermediate. Accordingly, we found that overexpression of the mutants cause cell-cycle arrest and dominant lethality. Our work identifies the OCM complex as competent for MCM2-7 dimerization, reveals MCM2-7 dimerization as a limiting step during pre-RC formation and defines critical mechanisms that explain how origins are licensed.

## INTRODUCTION

DNA replication requires a robust replication machinery to ensure faithful duplication of the genome before cell division. To ensure that each part of the genome is replicated only once, helicase loading and helicase activation is separated into two phases (1). During helicase loading, also known as DNA licensing, the six subunit origin recognition complex (ORC), Cdc6 and Cdt1 facilitate the loading of the mini-chromosome-maintenance 2–7 (MCM2-7) helicase onto DNA to form the prereplicative complex (pre-RC) (2). MCM2-7, composed of six highly related proteins, forms a hexamer in solution but is loaded as a double-hexamer onto DNA (3–5). The Mcm proteins form the core of the eukaryotic replicative helicase (6,7), but the MCM2-7 complex is inactive within the pre-RC (3,4). Helicase activation during S-phase is triggered by a number of protein factors, cyclin dependent kinase (CDK) and Dbf4 dependent kinase (DDK) that target the MCM2-7 complex (1).

Pre-RC assembly is an ATPase-dependent process (1). The pre-RC proteins Orc1-5, Cdc6 and Mcm2-7 belong to the AAA+ family of ATPases (8,9). In *Saccharomyces cerevisiae*, Orc1 and Cdc6 bind and hydrolyse ATP, while Orc5 can only bind ATP and Orc2-4 have completely lost the ability to bind ATP (9,10). Work over the past few years has shown that Cdc6 ATPase is required for MCM2-7 loading, suggesting that Cdc6 ATPase is an integral part of the MCM2-7 loading mechanism (10,11). On the other hand, it was recently found that Orc1 ATP hydrolysis is also required for Cdt1 release and MCM2-7 double-hexamer formation, indicating that both Orc1 and Cdc6 work in a synergistic manner (12). In contrast, the MCM2-7 ATPase activity is not required for pre-RC formation in *Xenopus* cell-free extracts and for *in vitro* pre-RC loading using purified proteins, which is based on mutants in the Walker A motif in *Xenopus* Mcm6, Mcm7 and the arginine finger of *S. cerevisiae* Mcm3 (12,13). Recently it was shown that in the absence of ATP hydrolysis an initial ORC/Cdc6/Cdt1/MCM2-7 complex is formed on origin DNA. This complex contains multiple Cdt1 molecules (14), but only a single MCM2-7 hexamer (15). A cryo electron microscopy structure of this complex has revealed the overall architecture of the complex and identified that the MCM2-7 C-termini interact with ORC/Cdc6 (16). On ATP hydrolysis, Cdt1 is released from the initial complex (10), resulting in an ORC/Cdc6/MCM2-7 (OCM) complex (12). However, the transition from OCM to MCM2-7 double-hexamer is a slow process (12). This indicates that origin recruitment of the second hexamer is more complex and mechanistically distinct from the recruitment of the first hexamer.

How the OCM is transformed into the double-hexamer is still elusive and so is the identity of many reaction intermediates during pre-RC assembly. Is the OCM complex proficient to directly recruit a second hexamer during initial MCM2-7 hexamer dimerization and what are the structural requirements for successful MCM2-7 double-hexamer formation are unresolved questions. Addressing these questions is necessary to uncover the mechanism of regulated pre-RC assembly and cell proliferation.

In this study, we investigated the process of MCM2-7 hexamer dimerization. We define MCM2-7 dimerization as the state when the two MCM2-7 hexamers are bound for the first time to the replication origin, but have not yet formed a salt stable double-hexamer. We show that a series of MCM2-7 double-hexamer interface mutants halt pre-RC formation at a late stage before double-hexamer formation. *In vivo* these mutants lead to G1 arrest or slow entry into S-phase and cause dominant lethality. Furthermore, we observed that the MCM2-7 mutants assembled into an OCM complex in the presence of ATP. Remarkably, the MCM2-7 interface mutants, once integrated into the OCM complex, were capable of MCM2-7 dimerization, but did not support salt stable double-hexamer formation. Interestingly, MCM2-7 dimerization is only occurring after ATP hydrolysis has occurred, highlighting that the OCM alone and not the initial ORC/Cdc6/Cdt1/MCM2-7 complex is able to form MCM2-7 dimers. These results suggest that the MCM2-7 structure changes on ATP hydrolysis and Cdt1 release to make the OCM competent for MCM2-7 dimerization. Furthermore, the data show that ORC/Cdc6 chaperones MCM2-7 before MCM2-7 double-hexamer formation. In summary, our data demonstrate that ORC/Cdc6 facilitates directly MCM2-7 dimerization and show that productive MCM2-7 dimerization is essential for pre-RC formation.

## MATERIALS AND METHODS

### *In vitro* pre-RC assembly assay

The pre-RCs were assembled in a one-step reaction: 40 nM ORC, 80 nM Cdc6, 40 nM Cdt1, 40 nM MCM2-7 wt or Ins mutants and 120 U Lambda phosphatase in buffer A (50 mM Hepes-KOH, pH 7.5, 100 mM KGlu, 10 mM MgAc, 50 μM ZnAc, 3 mM ATP, 5 mM DTT, 0.1% Triton and 5% Glycerol) plus 2 mM MnCl_2_ were added to 6 nM pUC19-ARS1 plasmid beads for 15 min at 24°C. Beads were washed three times with buffer A plus 1 mM EDTA or buffer B (50 mM Hepes-KOH, pH 7.5, 1 mM EDTA, 500 mM NaCl, 5% Glycerol, 0.1% Triton and 5 mM DTT) before digestion with 1U of DNaseI in buffer A plus 5 mM CaCl_2_ for 6 min at 24°C.

### Co-immunoprecipitation assays

Two standard size pre-RC reactions were prepared as described above (*in vitro* pre-RC assembly assay), released from DNA with AluI (New England Biolabs) for 7.5 min at 24°C, pooled, immunoprecipitated with anti-maltose binding protein (MBP) (New England Biolabs) or anti-Mcm2 antibody coupled to protein G beads for 7.5 min at 24°C, washed three times with 100 µl of buffer A and analyzed by western blot with anti-Mcm2, anti-Cdt1, anti-Cdc6 (9H8/5, Abcam, ab20150) and anti-Orc5.

## RESULTS

### Mutations in the MCM2-7 double-hexamer interface are dominant lethal and cause a cell cycle arrest

**Figure 1.**
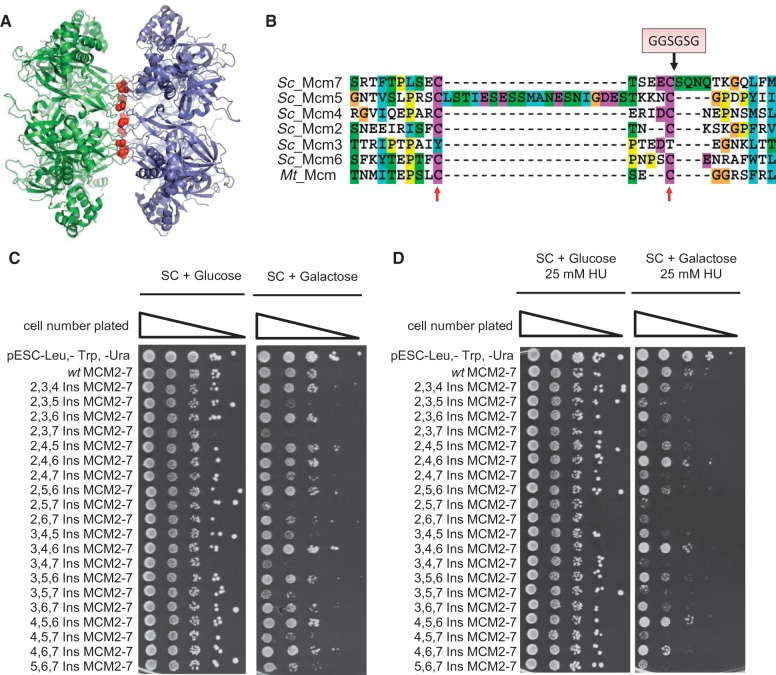
Mutations in the MCM2-7 hexamer interface are dominant lethal. (**A**) Crystal structure of the N-termini of mtMCM assembled into a double-hexamer (17). A conserved cysteine, which marks the site critical for hexamer interaction is shown in red. (**B**) Alignment of amino acids of the N-terminal domain of scMCM2-7 and mtMCM involved in zinc binding and the hexamer interface. Two conserved cysteines are marked with red arrows. A six amino acid loop (GGSGSG) was inserted after the second cysteine to specifically disturb the double-hexamer interface of MCM2-7. MCM2-7 hexamer-interface mutants or combinations were overexpressed (galactose) from 2-micron plasmids in the (**C**) absence or (**D**) presence of 25 mM HU to test for dominant lethal effects. In plates containing glucose the expression gets repressed. A strain containing vectors (pESC-Leu, pESC-Trp and pESC-Ura) and a strain expressing *wt* MCM2-7 served as a control. All tested MCM strains contained, in addition to the mutants, the complementing *wt MCM* genes so that always all six *MCM2-7* genes were overexpressed. Serial dilutions of cells ranging from 1 × 10^7^ to 1 × 10^3^ were spotted on selective SC plates.

MCM2-7 exists as a double-hexameric complex on DNA, both *in vivo* and *in vitro* (3–5). However, it is currently unclear how this complex is formed during pre-RC formation. To analyze the process, we generated a series of mutants within the MCM2-7 double-hexamer interface, which were designed to interfere with successful MCM2-7 double-hexamer formation. Our mutational approach is based on the crystal structure of an archaeal N-terminal Methanobacterium thermoautotrophicum MCM (mtMCM) (17) and a previous mutational approach used to interfere with mtMCM double-hexamer formation (18). The archaeal mtMCM and eukaryotic MCM2-7 are closely related in structure. In the mtMCM structure, the interface between the two hexamers spans a large surface and is localized within the zinc binding domain of the MCM family (Figure 1A). Fletcher *et al.* introduced a six amino acid insertion (6 Ins) between Cys-158 and Gly-159 of mtMCM, encoding a flexible GSGSGG sequence. mtMCM assembles into a homo-hexamer, generating a complex containing six mutant subunits in each hexamer. The mutant hexamer was not competent for double-hexamer formation *in vitro*, but had no influence on mtMCM hexamer-structure, ATPase or DNA binding activity. Because the *S. cerevisiae* MCM2-7 (scMCM2-7) complex is a hetero-hexamer, it is possible to engineer mutant complexes containing a scalable number of mutations from one to six. This strategy may allow the generation of mutant complexes with intermediate phenotypes.

To generate scMCM2-7 mutants analogous to the mtMCM 6ins mutants, we aligned mtMCM with scMCM2-7 (Figure 1B). Consequently, we created 6ins mutations in MCM2-7 by inserting the GSGSGG peptide sequence after the conserved cysteine (Figure 1B) in each of the six scMcm proteins (Ins MCM2-7) to generate a series of Ins mutants, which we also refer to as (double)-hexamer interface mutants, as the Ins site is located at the predicted MCM2-7 double-hexamer interface. As we predict that MCM2-7 double-hexamer formation is an important step during pre-RC formation, we set out to test whether these Ins mutants exhibit a dominant lethal phenotype *in vivo* (Figure 1C and D and Supplementary Figure S1). The wild-type (*wt*) and mutant *mcm* genes were co-expressed from 2-micron plasmids using galactose-inducible promoters to overexpress all six Mcm proteins. Overexpression was performed in a yeast strain containing the *wt* chromosomal copies of the *MCM2-7* genes. Whenever a single Ins mutant was overexpressed together with the five *wt* Mcm proteins, we observed no effect (Supplementary Figure S1A). The simultaneous overexpression of 2 Ins mutants—especially the combination of Ins Mcm5 and Ins Mcm7 with *wt* Mcm2, 3, 4 and 6 (5,7 Ins)—caused some dominant lethality (Supplementary Figure S1B). Co-overexpression of 3 (Figure 1C) or 4 Ins mutants (Supplementary Figure S1D) leads to a spectrum of phenotypes: from poor growth to *wt*-like growth. Overexpression of 5 or 6 Ins mutants showed a strong dominant-lethal phenotype in all cases (Supplementary Figure S1E). These results show that the effect of the mutants is additive, suggesting that a partial disruption of the MCM2-7 double-hexamer interface is tolerated. Nevertheless, not all strains expressing Ins mutant combinations showed the same degree of dominant lethality, suggesting that Mcm N-termini may have different roles during MCM2-7 double-hexamer formation.

To investigate if any of the Ins mutants influence cell viability during replication fork arrest, we challenged the Ins mutants with hydroxyurea (HU) (Figure 1D and Supplementary Figure S1). Overexpression of single Ins mutants in combination with the five *wt* Mcm proteins showed no HU-specific phenotype. This shows that no single-mutant causes a specific defect during fork arrest, and suggests that individual mutations do not compromise S-phase–specific functions of MCM2-7. Also, some triple mutants including 3,4,6 Ins and 4,5,6 Ins did not display dominant lethality and were not sensitive to HU. A number of mutants including 2,5,6 Ins and 3,6,7 Ins displayed near normal growth on overexpression, but were sensitive to HU. In contrast, the overexpression of 4,5,7 Ins and 5,6,7 Ins and others caused dominant lethality, which was further enhanced in the presence of HU. To judge these results, one has to take into account that multiple MCM2-7 double-hexamer complexes are loaded at each origin, but only one double-hexamer per origin is needed for DNA replication. The other MCM2-7 double-hexamer copies have a backup role and become activated in case a replication fork becomes blocked (19,20). Therefore, mutants that reduce MCM2-7 loading, but do not completely block pre-RC formation, should show a growth reduction when challenged by HU (Supplementary Figure S1), as these mutants do not have sufficient MCM2-7 complexes to generate new forks after terminal fork stalling.

**Figure 2.**
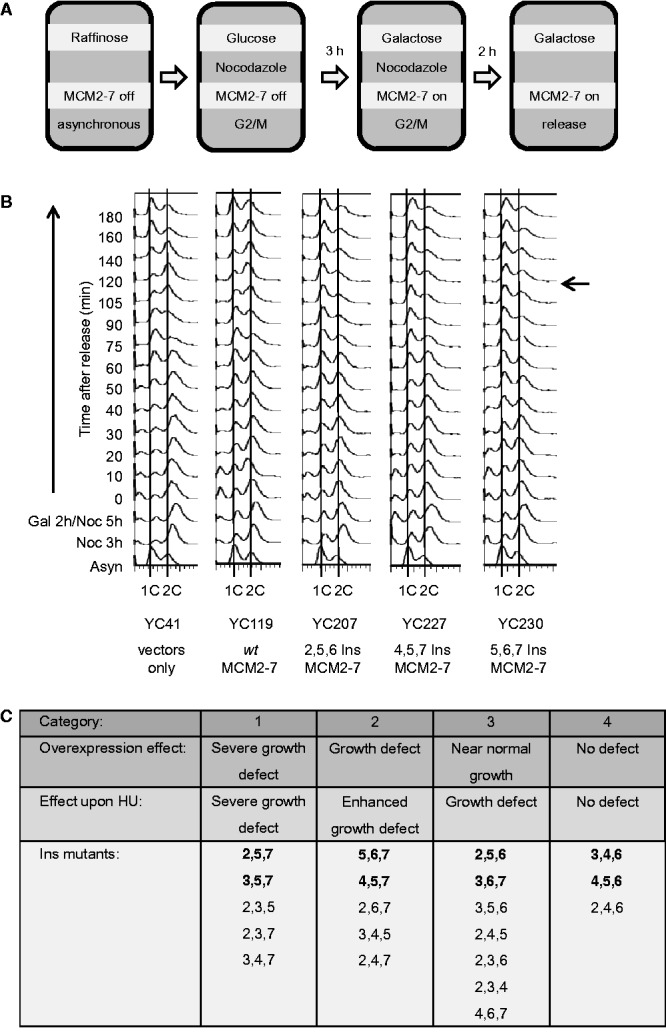
Overexpression of MCM2-7 hexamer-interface mutants lead to a cell cycle arrest. (**A**) Scheme indicating the course of the experiment. Expression of *MCM2-7* genes or distinct *mcm2-7* hexamer-interface mutant combinations is initially repressed by raffinose in the media. Vectors lacking the *MCM* genes and vectors encoding *wt MCM2-7* genes served as controls. Nocodazole was added to the media to arrest the cells at G2/M-phase. Three hours later protein expression was induced by the addition of galactose. After expression for 2 h, the cells were washed and released from the G2/M block while maintaining protein expression from the GAL promoters. (**B**) Flow cytometry of five yeast strains progressing from G2/M into the cell cycle. Two strains serve as negative controls: YC41 (empty vectors) and YC119 (*wt* MCM2-7). Cell cycle arrest in G1 was observed at 120 min (marked with an arrow) after G2 release when the 2,5,6 Ins MCM2-7, 4,5,7 Ins MCM2-7 and 5,6,7 Ins MCM2-7 proteins were overexpressed. (**C**) Categories for the triple Ins mutants and summary of the growth phenotypes observed. The mutants printed in bold have been purified and analyzed for their ability to form a pre-RC complex.

To address whether overexpression of triple mutants, with a near normal (2,5,6 Ins) to intermediate growth phenotype (4,5,7 Ins and 5,6,7 Ins), alters cell cycle progression, we arrested cells with nocodazole in G2 phase of the cell cycle, induced MCM Ins mutant expression, then released the cells and monitored cell cycle progression using flow cytometry (Figure 2A). All the Ins mutants tested caused an arrest at the G1/S boundary or slow G1-S transition with a near 1C DNA content (Figure 2B, compare the 120-min time points). In contrast, the corresponding strain overexpressing *wt* MCM2-7 progressed normally through the cell cycle. It is important to note that in the case of the 2,5,6 Ins mutant we observed hardly any growth defect on solid media, although we detected a cell cycle defect for the same mutant in liquid culture. This could be explained by the fact that galactose promoter-induced expression is stronger for yeast grown in liquid media than for yeast grown on solid media. Also, the overall growth rate of cells on solid media is much lower than the growth rate of yeast in liquid culture, giving yeast growing in a petri dish more time to compensate for a cell cycle defect.

In summary, we found that the triple mutants could be grouped into four categories (Figure 2C) of phenotypes. The first category, which includes among others 2,5,7 Ins and 3,5,7 Ins, displayed strong dominant lethality in the absence and presence of HU. The second category, which includes 4,5,7 Ins and 5,6,7 Ins, displayed dominant lethality on overexpression and this effect was further enhanced by HU. The third category, including 2,5,6 Ins and 3,6,7 Ins, displayed near-normal growth on overexpression, but dominant lethality in the presence of HU. Finally, the fourth category, including 3,4,6 Ins and 4,5,6 Ins, did not cause dominant lethality on overexpression and was not sensitive to HU. Furthermore, this analysis indicates that a mutation in Mcm7 more frequently causes a strong growth defect, while mutations in Mcm4 and Mcm6 have the least effect.

Based on this work, it is clear that Ins mutations can cause a defect in cell cycle progression and viability. To discover if these mutants have a specific pre-RC formation defect, we used them in the following several *in vitro* assays.

### MCM2-7 double-hexamer interface mutants are defective in pre-RC assembly

**Figure 3.**
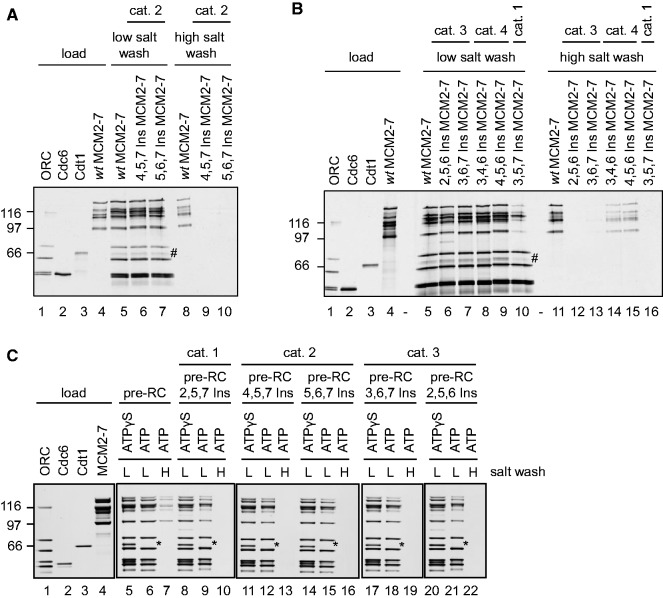
MCM2-7 interface mutants block MCM2-7 loading. (**A** and **B**) pre-RC assays to test the ability of MCM2-7 hexamer-interface mutants to associate with origin DNA in the presence of ATP, ORC, Cdc6 and Cdt1 and to load in a salt-stable way onto DNA. The hash symbol indicates the presence of a nonspecific band that overlaps with the Cdt1 band. (**C**) Similar as A, but in lanes labeled ATPγS the ATP hydrolysis was blocked, to verify the ability of the mutants to form an initial ORC/Cdc6/Cdt1/MCM2-7 complex. The asterisk marks the absence of Cdt1 in the sample containing ATP, where ATP hydrolysis–dependent Cdt1 release and OCM formation is occurring. For all assays (A–C) a reconstituted system containing purified 40 nM ORC, 80 nM Cdc6, 40 nM Cdt1, 40 nM MCM2-7 or 40 nM MCM2-7 mutants and origin containing 6 nM pUC19-ARS1 DNA coupled to magnetic beads was used to test the ability of the proteins to load the MCM2-7 helicase onto DNA. Experiments are shown with a 10% load (A and B) or a 30% load (C) of *wt* ORC, Cdc6, Cdt1 and MCM2-7. Experiments labeled with ‘low salt’ or ‘L’ tested *wt* MCM2-7 and several mutant combinations for their ability to associate with origin containing DNA in the presence of ORC, Cdc6 and Cdt1. Experiments labeled with ‘high salt’ or ‘H’ were performed in the same manner as low-salt experiments, but in the end were challenged with high salt, which removes ORC, Cdc6, Cdt1 and in addition a fraction of the MCM2-7 helicase that was not loaded onto DNA.

Our *in vitro* analysis concentrated on triple mutants, as these displayed a wide range of *in vivo* phenotypes with some showing strong dominant lethality while others were unaffected. We tested a number of triple mutants, two from each category (Figure 2C, marked in bold), for their ability to participate in pre-RC formation. Firstly, we purified the Ins mutants using HA affinity chromatography and gel filtration. The mutant proteins fractionated during the gel-filtration step similar to *wt* MCM2-7 (Supplementary Figure S2), besides one category 1 mutant (3,5,7 Ins) that displayed reduced hexamer stability. These data show that most Ins mutants are capable of forming a hexameric complex. Then we tested the mutants in an *in vitro* pre-RC assay, which uses purified ORC, Cdc6, Cdt1 and MCM2-7 proteins and origin DNA linked to magnetic beads. We analyzed two category 2 MCM mutants (4,5,7 Ins and 5,6,7 Ins) (Figure 3A) and found that these formed complexes with ORC/Cdc6 similarly to *wt* MCM2-7 (Figure 3A, lanes 5–7). To identify the presence of Cdt1 more clearly we performed also western blots (Supplementary Figure S3). A high-salt wash of pre-RC complexes removes DNA-associated proteins like ORC, Cdc6 and Cdt1, but retains loaded MCM2-7, as it encircles DNA as a double-hexameric ring (3,4). As expected, we found that *wt* MCM2-7 was loaded onto DNA, as it was retained on the DNA after a high-salt wash (Figure 3A, lane 8). In contrast, the 4,5,7 Ins and 5,6,7 Ins MCM2-7 mutants failed to form a salt-stable MCM2-7 complex with DNA (Figure 3A, lanes 9 and 10), suggesting that these MCM mutants were not loaded onto DNA. Next we tested two mutants of category 3 (2,5,6 Ins, 3,6,7 Ins) and 4 (3,4,6 Ins, 4,5,6 Ins) and one mutant of category 1 (3,5,7 Ins) in the same assay. Most of the mutants associated with DNA under low-salt buffer conditions similar to *wt* MCM2-7 (Figure 3B, lanes 5–10). However, the category 1 (3,5,7 Ins) mutant, which exhibited reduced complex stability during the purification (Supplementary Figure S2), associated less efficiently with ORC/Cdc6 than *wt* MCM2-7 (Figure 3B, compare lane 5 and 10). For this reason we excluded this mutant from other studies. The category 4 mutants displayed some salt stable MCM2-7 loading (Figure 3B, compare lane 14 and 15 to lanes 11) and the two category 3 mutants studied blocked the loading of salt-stable MCM2-7 (Figure 3B, lanes 12 and 13). In summary, the mutants tested belonging to category 1, 2 and 3 blocked salt-stable MCM2-7 loading. On the other hand, the two category 4 mutants affected MCM2-7 double-hexamer formation, but did not completely block this process. This is consistent with our observation that these mutants do not cause dominant lethality. Because the category 4 mutants (3,4,6 Ins 4,5,6 Ins) did not completely arrest pre-RC formation, we excluded these mutants from other assays, as they could produce weak or ambiguous phenotypes. Furthermore, these results indicate that MCM2-7 with three mutated subunits can retain some functionality. It is clear from these experiments that all, but one, MCM2-7 double-hexamer interface mutant tested associated well with ORC/Cdc6, and that the category 2 and 3 Ins mutants failed to form a salt-stable MCM2-7 complex onto DNA.

### MCM2-7 hexamer interface mutants release Cdt1 efficiently during pre-RC formation

During pre-RC formation an ORC/Cdc6 complex assembles on DNA, which then recruits Cdt1/MCM2-7 (21,22) to form an initial ORC/Cdc6/Cdt1/MCM2-7 complex (14,15). On ATP hydrolysis and Cdt1 release this complex is transformed into the OCM (12). To test if the triple Ins mutants assemble into an initial ORC/Cdc6/Cdt1/MCM2-7 complex and release Cdt1 (Figure 3 C) we compared complex formation in the presence of ATPγS and ATP with several Ins mutants belonging to categories 1–3. In the presence of ATPγS, which can be only slowly hydrolysed, we observed efficient binding of ORC, Cdc6, Cdt1 and MCM2-7 to origin DNA both with *wt* and Ins MCM2-7. In the presence of ATP we detected ORC, Cdc6 and MCM2-7, but Cdt1 was absent (Figure 3C, marked with an asterisk). Reactions with ATP that were high-salt washed showed salt-resistant MCM2-7 in the case of *wt* MCM2-7, but not in the cases of category 1, 2 and 3 Ins mutants. These data indicate that the Ins mutants form an ‘initial’ ORC/Cdc6/Cdt1/MCM2-7 complex in the presence of ATPγS. Furthermore, we observed that all Ins mutants tested released Cdt1 in the presence of ATP, suggesting that these mutants are capable of OCM complex formation.

Our *in vitro* analysis indicated that category 1 (2,5,7 Ins), 2 (4,5,7 Ins, 5,6,7 Ins) and 3 mutants (2,5,6 Ins and 3,6,7 Ins) associated efficiently with ORC/Cdc6, but failed to produce salt-stable MCM2-7 loading. Out of the six mutants that blocked MCM2-7 double-hexamer formation, the 2,5,6 Ins mutant caused the least dominant lethal effect (Figures1 and 2C). As we were interested in a mutant that blocks MCM2-7 loading, but besides this causes minimal defects, our further *in vitro* work was mostly focussed on the 2,5,6 Ins mutant.

### The 2,5,6 Ins MCM2-7 hexamer interface mutant arrests after OCM formation

**Figure 4.**
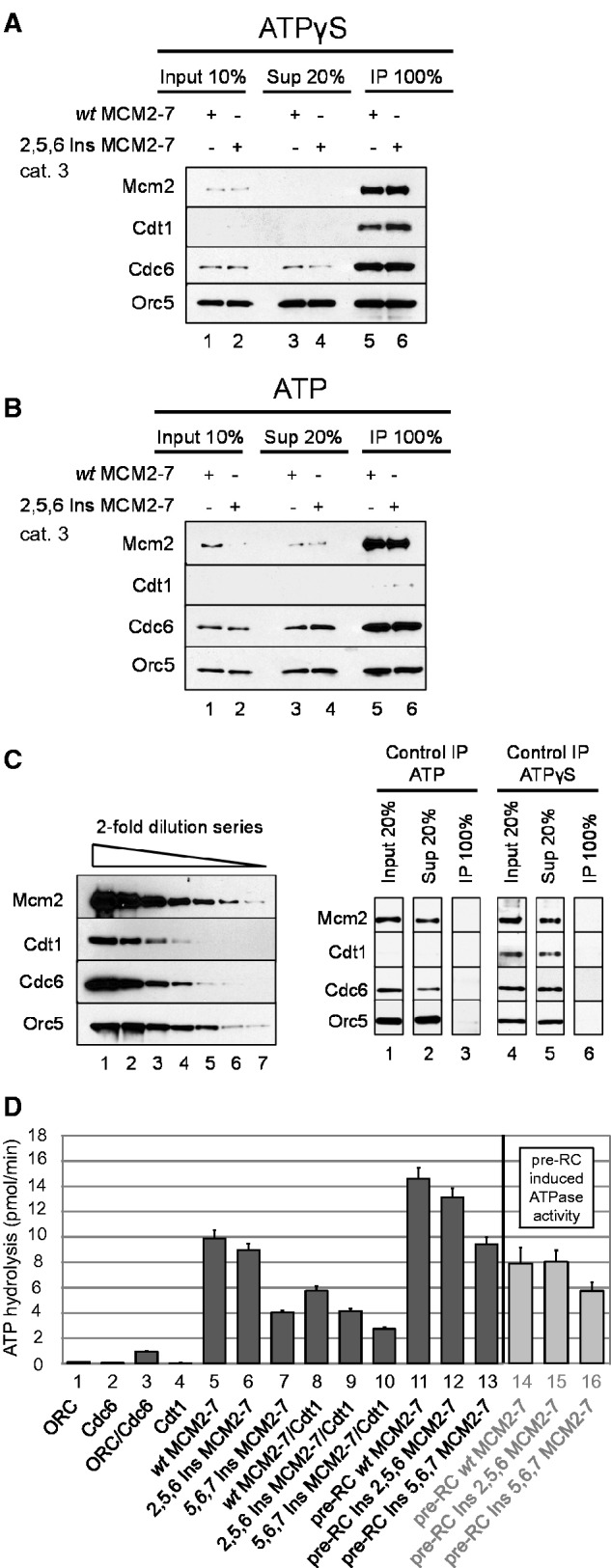
MCM2-7 hexamer interface mutants arrest during pre-RC formation in complex with ORC/Cdc6. (**A**) Pre-RC complexes with *wt* MCM2-7 and 2,5,6 Ins MCM2-7 were formed in the presence of ATP and ATPγS (**B**) and released from magnetic beads via restriction endonuclease digest. Complexes were immunoprecipitated with anti-Mcm2 antibodies, analyzed by western blot and compared with a 2-fold dilution series of pre-RC proteins and a control IP (**C**). (**D**) Analysis of ATPase activities during pre-RC assembly with *wt* MCM2-7, 2,5,6 Ins MCM2-7 and 5,6,7 Ins MCM2-7. The ATP hydrolysis rates were determined for the indicated proteins in the presence of ARS1 origin DNA. Together ORC, Cdc6, Cdt1 and MCM2-7 induced a strong ATPase activity. Lanes 14–16 show only the pre-RC–induced ATPase activity [ATPase activity of the full pre-RC reaction with the ATPase activity of the individual components (ORC/Cdc6 + Cdt1/MCM2-7) subtracted].

The pre-RC assay suggested that 2,5,6 Ins and other mutants were capable of releasing Cdt1 in an ATP hydrolysis–dependent manner. To corroborate these results we determined the ability of the 2,5,6 Ins category 3 mutant to form ORC/Cdc6/Cdt1/MCM2-7 (Figure 4A) and OCM (Figure 4B) complexes by immune precipitation (IP) and in parallel we performed the same experiment with *wt* MCM2-7. To measure ‘initial’ ORC/Cdc6/Cdt1/MCM2-7 complex formation we assembled reactions in the presence of ATPγS, washed the sample and then released protein–DNA complexes from the magnetic beads with a restriction enzyme. To avoid coprecipitation of separate complexes, we used the AluI restriction enzyme, which introduces 16 cuts into the plasmid of 2880 bp and excises an origin containing fragment of 237 bp. Then we immunoprecipitated 2,5,6 Ins MCM2-7 or *wt* MCM2-7 containing complexes with a Mcm2 antibody. The experiments were analyzed by quantitative western blotting and a 2-fold dilution series of pre-RC proteins is shown in Figure 4C. The IP showed efficient co-precipitating of Orc5, Cdc6, Cdt1 with Mcm2 for both *wt* and 2,5,6 Ins MCM2-7 (Figure 4A, lanes 5 and 6). This shows that the 2,5,6 Ins mutant forms an ‘initial’ ORC/Cdc6/Cdt1/MCM2-7 complex in the presence of ATPγS in a similar way to *wt* MCM2-7. However, with ATP we observed for both *wt* and 2,5,6 Ins MCM2-7, Orc5 and Cdc6 co-precipitation with Mcm2 (Figure 4B, lanes 5 and 6), but Cdt1 was absent. This shows that both 2,5,6 Ins MCM2-7 and *wt* MCM2-7 form an OCM complex. In summary, these data demonstrate that 2,5,6 Ins MCM2-7 mutant can assemble into an ‘initial’ ORC/Cdc6/Cdt1/MCM2-7 complex, which then matures on ATP hydrolysis and Cdt1 release into the OCM complex. To determine whether the 2,5,6 Ins mutant does indeed promote ORC/Cdc6 ATPase activity during OCM formation, we measured ATP hydrolysis in the context of the pre-RC assay (12).

We found that 2,5,6 Ins MCM2-7 on its own hydrolyzed ATP at a similar level than *wt* MCM2-7 (Figure 4D, lanes 5 and 6). In context of Cdt1, the ATP hydrolysis rate of 2,5,6 Ins MCM2-7 was reduced, consistent with Cdt1/2,5,6 Ins MCM2-7 complex formation (Figure 4D, lane 9). Interestingly, *wt* MCM2-7/Cdt1 or 2,5,6 Ins MCM2-7/Cdt1 addition to ORC/Cdc6 increased the ATP hydrolysis rate significantly (Figure 4D, lane 11 and 12), which is consistent with induction of ATP hydrolysis during pre-RC formation (Figure 4D, lanes 14 and 15) (12). We define the ‘pre-RC–induced ATPase activity’ as the ATPase activity of the full pre-RC reaction with the ATPase activities of the individual components (ORC/Cdc6 + Cdt1/MCM2-7) subtracted (Figure 4D, marked in light gray). This indicates that the 2,5,6 Ins mutant is inducing ATP hydrolysis during pre-RC formation in a similar fashion to *wt* MCM2-7.

To understand if a category 2 mutant behaved similarly, we repeated the ATPase assay with 5,6,7 Ins (Figure 4D). Interestingly, with this mutant on its own we observed 60% reduced ATPase activity. An ATP hydrolysis defect in general should have no influence on pre-RC formation, as MCM2-7 ATPase is not required for pre-RC formation (12,13). However, it appears possible that the 5,6,7 Ins mutant may have a conformational alteration, which could impact on its function. We observed with the 5,6,7 Ins mutant a pre-RC–induced ATPase activity that was reduced by 25% in comparison with the *wt*. This reduction could result in slower OCM formation, but is not blocking Cdt1 release in general (Figure 3A and 3C). Nevertheless, it is clear that the 5,6,7 Ins category 2 mutant has a conformational alteration, which leads to reduced MCM2-7 and pre-RC–induced ATP hydrolysis. On the other hand, we demonstrate that the 2,5,6 Ins category 3 mutant is able to assemble into an ‘initial’ ORC/Cdc6/Cdt1/MCM2-7 complex, to facilitate normal pre-RC–induced ATP hydrolysis, to release Cdt1 and to form an OCM complex.

### The transition from MCM2-7 dimer to double-hexamer makes the helicase competent for efficient DDK phosphorylation

**Figure 5.**
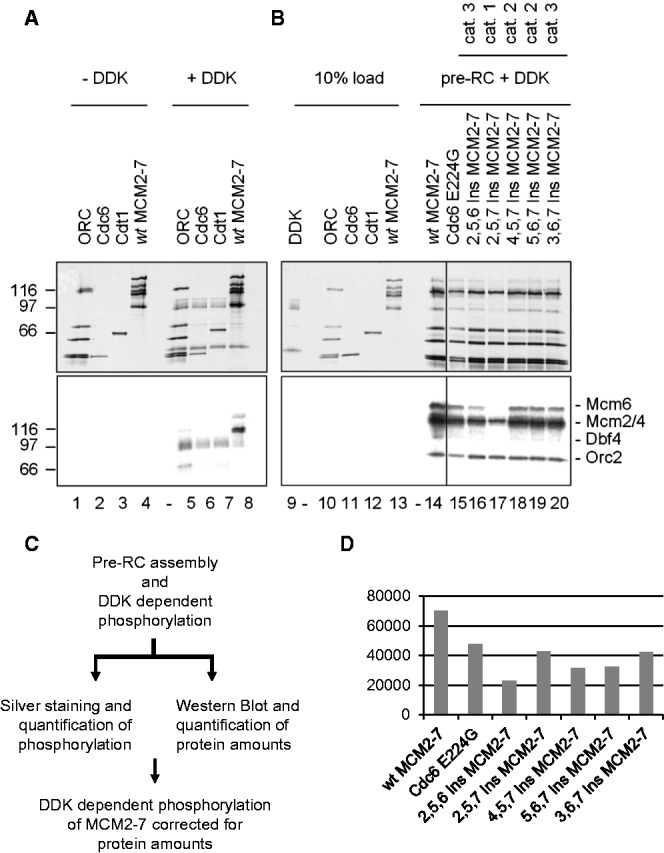
Double-hexamer formation triggers a structural change in MCM2-7. DDK-mediated phosphorylation of individual pre-RC proteins (lanes 1–8) or DNA-bound pre-RC complexes (lanes 9–20). The upper panel shows silver stained gels and the lower panel the autoradiogram of the same gel. Forty nanomolar ORC, Cdc6, Cdt1 and wild-type MCM2-7 were individually incubated for 15 min at 27°C either in the absence (lanes 1–4) or in the presence of 40 nM DDK and γ^32^P-ATP (lanes 5–8). The pre-RC assembly was performed with 6 nM pUC19-ARS1, 40 nM ORC, 80 nM Cdc6 wild-type or Cdc6 E224G, 40 nM Cdt1 and 40 nM wild-type MCM2-7 or 40 nM Ins MCM2-7 mutants (lanes 14-–20). A 10% load of the unphosphorylated proteins and DDK is shown in lanes 9–13. The pre-RC was formed with wild-type pre-RC proteins (lane 14), a Cdc6 ATP hydrolysis mutant E224G (lane 15) and five different Ins MCM mutants (lane 16–20).

Our data show some triple mutants–displayed defects in OCM formation. On the other hand, the 2,5,6 Ins mutant appeared capable of normal ATP hydrolysis–driven Cdt1 release, suggesting that this mutant could function for processes after OCM complex formation (Figures 3 and 4). MCM2-7, within the OCM, might adopt a structure similar to the double-hexamer. To address this question, we used DDK kinase (Cdc7-Dbf4) as a structural sensor, as DDK has been shown to preferentially phosphorylate MCM2-7 within the double-hexamer and less efficiently if pre-RC assembly is blocked by a Cdc6 E224G ATP hydrolysis mutant (23,24). Consistent with previous work, we found that purified MCM2-7 in the absence of other proteins is weakly phosphorylated by DDK on Mcm2, Mcm4 and Mcm6 (Figure 5A, lane 8). Interestingly, the purified Ins mutants were phosphorylated by DDK in an identical manner to *wt* MCM2-7 (Supplementary Figure S4). This result indicates that the 6 Ins mutations did not result in a change of the MCM2-7 complex that leads to altered phosphorylation. To evaluate if the Ins containing OCM is targeted by DDK in a similar manner than the MCM2-7 double-hexamer we prepared pre-RC reactions with *wt* MCM2-7 and several Ins mutants. After complex formation, DDK and P^32^-ATP were added and then the reactions were split and analyzed for MCM2-7 protein content (Supplementary Figure S5) and for DDK-dependent phosphorylation of Mcm and other proteins (Figure 5B and C). After correcting for differences in protein amounts (Supplementary Figure S5) we plotted the DDK-dependent phosphorylation of *wt* MCM2-7 and the Ins mutants in Figure 5D. The data show robust phosphorylation of loaded MCM2-7 and reduced phosphorylation in the presence of Cdc6 E224G, which partially blocks pre-RC assembly. With the Ins mutants we also observed reduced phosphorylation of Mcm2, Mcm4 and Mcm6 (Figure 5B and D). These results indicate that Ins MCM2-7 mutants, when integrated in the OCM, are not an optimal substrate for phosphorylation by DDK, similar as with Cdc6 E224G. Another possibility could be that the Ins mutants are differentially pre-phosphorylated, which could affect their ability to be phosphorylated by DDK (23). Although this is possible, we think that it is unlikely, as DDK phosphorylated purified *wt* and mutant MCM2-7 in an identical fashion.

### The OCM complex is competent for MCM2-7 dimerization

**Figure 6.**
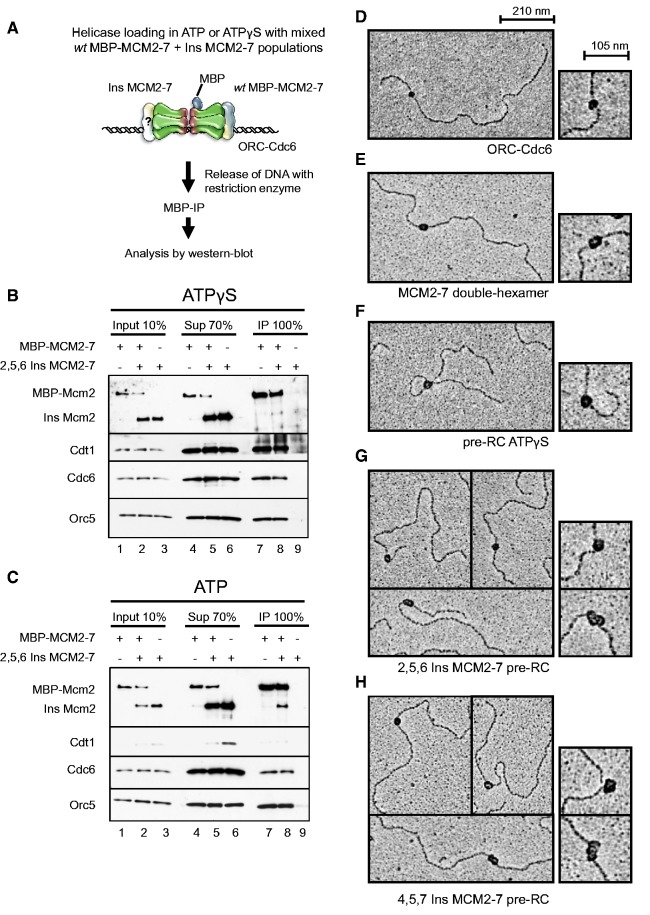
MCM2-7 hexamer-interface mutants promote ATP hydrolysis–dependent MCM2-7 dimerization. (**A**) Experimental outline for (**B** and **C**). Pre-RC assays were assembled in the presence of ATPγS and ATP. When tagged and untagged MCM2-7 were used, equimolar amounts of each complex were combined in pre-RC reactions. Complexes were released from DNA via restriction digest and DNA-bound complexes were immunoprecipitated (IP) and analyzed by western blotting together with input and supernatant (Sup) using anti-Mcm2, anti-Cdt1, anti-Cdc6 and anti-Orc5 antibodies. (B) In the presence of ATPγS MCM2-7, dimerization is absent. The experiment was performed as described in (A). (C) MCM2-7 dimerization is facilitated by ATP hydrolysis. The experiment was performed as described in (B), except that ATP was used. (**D**) ORC/Cdc6, (**E**) MCM2-7 double-hexamer, (**F**) pre-RC ATPgS (**G**) 2,5,6 Ins MCM2-7 or (**H**) 4,5,7 Ins MCM2-7 were assembled on DNA, gel-filtered, fixed with glutaraldehyde and visualized by rotary shadowing with platinum. Magnification of individual complexes is shown to indicate the differences in size and shape of ORC/Cdc6, MCM2-7 double hexamer and the 2,5,6 Ins and 4,5,7 Ins MCM2-7 containing complexes.

Currently it is unclear how the OCM is transformed into the MCM2-7 double-hexamer. One possibility is that the OCM itself functions as a platform for the double-hexamer (12). If that is the case, then a salt unstable MCM2-7 dimer could be formed before establishment of an MCM2-7 double-hexamer; however, such an MCM2-7 dimer has never been observed. Interestingly, the 2,5,6 Ins mutant supports normal OCM formation, but is unable to form a salt-stable MCM2-7 double-hexamer. Therefore we set out to investigate if the OCM formed with 2,5,6 Ins is competent to establish an MCM2-7 dimer. To measure dimerization, we used a co-immunoprecipitation approach using a mixture of MBP-tagged *wt* MCM2-7 with untagged 2,5,6 Ins MCM2-7 (category 3). Complexes were formed on DNA, released via restriction digest and immunoprecipitated with an anti-MBP antibody (Figure 6A). In our *in vitro* system 1–1.5 MCM2-7 molecules bind on average to each DNA molecule, indicating that each DNA plasmid could be in complex with more than one MCM2-7 hexamer. To hinder the coprecipitation of two independent OCM complexes, we cut the plasmid with the AluI restriction enzyme at 16 places and generating an origin containing fragment of 237 bp. Importantly, we performed the experiment in the presence of ATPγS as well to control for nonspecific dimerization, as in the presence of ATPγS only a single MCM2-7 hexamer is recruited to ORC/Cdc6 and Cdt1 (15). In the presence of ATPγS, we observed only negligible amounts of untagged Mcm2 in the anti-MBP pull-down (Figure 6B, lane 8), but efficient co-precipitation of Orc5, Cdc6 and Cdt1 with MBP-Mcm2. Remarkably, in the presence of ATP we observed robust co-immunoprecipitation of untagged MCM2-7 in the MBP-pulldown (Figure 6C, lane 8). Furthermore, this complex contained Cdc6 and Orc5, but Cdt1 was absent. To verify that MCM2-7 dimer is not involving two independent complexes held together by DNA, we repeated the experiment, but released the complexes by DNase I from the magnetic beads. We observed a similar dimer as with the Alu I restrictions digest (Supplementary Figure S6A). Importantly, the observed dimer does not represent a MCM2-7 double-hexamer, as *wt* MCM2-7 does not facilitate the loading of the 2,5,6 Ins MCM2-7 mutant into a salt stable MCM2-7 double-hexamer (Supplementary Figure S6C). Furthermore, MBP-MCM2-7 loads efficiently onto DNA (Supplementary Figure S6B and C). This experiment shows that a 2,5,6 Ins containing OCM complex is competent for MCM2-7 dimerization, whereas the initial ORC/Cdc6/Cdt1/MCM2-7 did not facilitate dimer formation.

To corroborate this result, we wanted to visualize such a dimer containing complex by electron microscopy using category 2 and 3 mutants. We studied metal shadowed pre-RC complexes containing Ins MCM2-7 and included ORC/Cdc6 (Figure 6D), *wt* MCM2-7 double-hexamer (Figure 6E) and a pre-RC arrested with ATPγS (Figure 6F) as controls. The pre-RC formed in the presence of ATPγS is a good reference, as it contains only a single MCM2-7 hexamer in complex with ORC/Cdc6 and Cdt1 (15). Interestingly, 2,5,6 Ins and 4,5,7 Ins MCM2-7 assembled in the presence of ATP in two species of complexes (Figure 6G and H). We observed a small Ins MCM2-7 containing complex (Figure 6F and G; upper part), which was similar to the pre-RC ATPγS complex. In addition, we observed a large Ins MCM2-7 containing complex (Figure 6F and G; lower part), which appeared bigger than the MCM2-7 double-hexamer, suggesting that this complex contains an MCM2-7 dimer in complex with ORC/Cdc6. Furthermore, we found that the small 2,5,6 Ins complex (77 out of 107 molecules, 71%) was more abundant than the large 2,5,6 Ins complex (30 out of 107 molecules, 28%). This is reminiscent of our finding that only some of the 2,5,6 Ins containing OCM complex was competent for dimerization. These electron microscopy data are consistent with the co-immunoprecipitation experiments and suggest that 2,5,6 Ins and 4,5,7 Ins can assemble into large MCM2-7 dimers in complex with ORC/Cdc6.

## DISCUSSION

Here we report that 2,5,6 Ins MCM2-7 can form an OCM complex in an ATP hydrolysis–dependent manner. Remarkably, this 2,5,6 Ins MCM2-7 containing OCM complex is competent for the initial recruitment of a second MCM2-7 hexamer and thus promotes MCM2-7 dimerization. However, the Ins mutants cannot form a MCM2-7 double-hexamer and MCM2-7 dimerization is not occurring in the absence of ATP hydrolysis. The MCM2-7 dimer, in contrast to the MCM2-7 double-hexamer, is a late pre-RC intermediate as it is found in complex with ORC/Cdc6 and is salt sensitive. These results suggest that ATPase-dependent OCM formation makes MCM2-7 competent for dimerization and that the 2,5,6 Ins mutation blocks the formation of a stable double-hexamer interface.

### The MCM2-7 hexamer interface mutation causes a late arrest in pre-RC formation

We have used MCM2-7 interface mutants to understand the mechanism of double-hexamer formation. The specific mutations were designed based on structural information of a close homologue, an archaeal mtMCM, and are localized toward the N-terminal tip of the protein complex. The same mutation in three scMCM subunits did not affect the purification characteristics of six category 2–4 Ins MCM2-7 mutants, the hexameric structure of these Ins MCM2-7 complexes as judged by gel filtration, the ability of most of the mutants to interact with ORC/Cdc6/Cdt1 or the DDK phosphorylation patterns of MCM2-7. Furthermore, we specifically found that the 2,5,6 Ins mutant assembles into an OCM complex and promotes pre-RC–induced ATP hydrolysis similar to *wt* MCM2-7. Based on these results, it is unlikely that the 2,5,6 Ins mutant has a large overall conformational alteration. Consistent with that, the 2,5,6 Ins MCM2-7 interface mutant is capable of MCM2-7 dimerization, but fails to form a salt-stable MCM2-7 double-hexamer. Within the ORC/Cdc6/Cdt1/MCM2-7 complex, ORC/Cdc6 interacts with the MCM2-7 C-termini (16), which leaves the MCM2-7 N-termini available for interaction. Thus the OCM recruits most likely the second MCM2-7 hexamer via an interaction involving the N-terminal hexamer interface. In case of the 2,5,6 Ins mutant, we predict that the partial contacts between the two MCM2-7 hexamers are strong enough to support recruitment of the second hexamer (dimerization), but not strong enough to allow salt-stable double-hexamer formation. On the other hand, it has been suggested that the second MCM2-7 could also be recruited via ORC/Cdc6 (14); however, the cryo-electron microscopy structure of the ORC/Cdc6/Cdt1/MCM2-7 did not identify a second ORC/Cdc6–MCM2-7 interaction site (16). Therefore we propose that initial hexamer dimerization occurs via the N-terminal hexamer interface that is followed by a structural change, which could involve a locking mechanism that facilitates the formation of a tight interaction between each MCM2-7 hexamer. The 2,5,6 Ins hexamer-interface mutant could block this process specifically, but we can not exclude the possibility that a different structural change within the N-terminal section could prevent stable double-hexamer formation. Another interesting possibility is that double-hexamer formation is coordinated with MCM2-7 ring opening, which may require orchestrated interactions between both hexamers.

Furthermore, other mutants may have additional defects, for example, 3,5,7 Ins blocks the association with ORC/Cdc6 (Figure 3B). One possibility is that the Ins mutation could alter the inter-subunit interactions, which could explain the ATP hydrolysis defect seen in 5,6,7 Ins.

Moreover, the MCM2-7 double-hexamer does not bind ORC/Cdc6 (15), but we observed that the MCM2-7 dimer maintains association with ORC/Cdc6, suggesting that successful MCM2-7 double-hexamer formation triggers another structural change in MCM2-7, resulting in ORC/Cdc6 release.

### Requirements for productive MCM2-7 dimerization

Previous work has shown that the binding of Cdt1/MCM2-7 to ORC/Cdc6 yields an initial ORC/Cdc6/Cdt1/MCM2-7 complex (14–16). In the absence of ATP hydrolysis no MCM2-7 dimerization occurs (15). Furthermore, Cdc6 and Orc1 ATP hydrolysis promotes the release of Cdt1 and yields an OCM complex. Consistent with that we found no dimerization of the 2,5,6 Ins category 3 MCM2-7 hexamer interface mutant in the presence of ATPγS. On the other hand, we detected dimerization with the same mutant in the presence of ATP using a co-immunoprecipitation assay, and we also observed complexes that appeared larger than the MCM2-7 double-hexamer by electron microscopy. Importantly, MCM2-7 does not form dimers in solution, showing that ORC/Cdc6 induces a dimerization competent state. Interestingly, the percentage of MCM2-7 dimer formation (27.6%) (Figure 6C) and MCM2-7 loading into double-hexamers (15–30%) (3) is similar. These data suggest that the MCM2-7 dimer is a precursor of the MCM2-7 double-hexamer. Moreover, the MCM2-7 dimer could represent a limiting step during pre-RC formation, since the majority of MCM2-7 molecules formed OCM complexes, but only some formed MCM2-7 dimers and double-hexamers.

In summary, our data show two requirements for initial MCM2-7 dimerization and dimer function during double-hexamer formation. The first requirement is OCM formation, as dimer establishment requires MCM2-7 to be in complex with ORC/Cdc6, e.g. MCM2-7 on its own or when integrated within the initial ORC/Cdc6/Cdt1/MCM2-7 complex does not form a dimer. The second requirement is the establishment of a tight MCM2-7 hexamer–hexamer interface, as Ins mutations do not block dimerization, but fail to form a double-hexamer.

Based on previous work (12) and this work, it is clear that ORC/Cdc6 maintains interaction with MCM2-7 after OCM formation. This indicates that pre-RC formation does not involve an uncomplexed single-hexamer intermediate or MCM2-7 dimer and we suggest that ORC/Cdc6 chaperones MCM2-7 hexamer before double-hexamer formation. The function of ORC/Cdc6 is likely to stabilize MCM2-7 on DNA and to hinder the loading of uncomplexed single-hexameric onto DNA. Importantly, our data suggest that OCM-induced MCM2-7 dimerization promotes stable MCM2-7 double-hexamer formation.

In the future, it will be important to determine (i) if one or two ORC/Cdc6 complexes participate in dimerization; (ii) if DNA sequence influences dimerization and (iii) to understand the structure of the dimeric MCM2-7 complex.

### An unequal role of the Mcm2-7 N-termini in double-hexamer formation

We have analyzed all combinations of three mutant *mcm* genes with three *wt MCM* genes for dominant lethal phenotypes and observed reduced growth with several mutants when compared with the overexpression of *wt* MCM2-7. Furthermore, for 2,5,6 Ins, 4,5,7 Ins and 5,6,7 Ins, we detected a cell cycle defect. This indicates that the Ins mutation interferes with an important function. Our *in vitro* analysis suggests that the 2,5,6 Ins mutant still maintains the capability for initial MCM2-7 dimerization, while this mutant fails to form the salt-stable MCM2-7 double-hexamer. This suggests that MCM2-7 double-hexamer formation is an essential process *in vivo*. When we compared the growth phenotype of the mutant combinations we found that the introduction of the Ins mutations in *mcm4* and *mcm6* affects growth weakly, but mutant combinations containing the *Ins-mcm7* mutation were more strongly affected. This could be due in part to a structural change, as the Ins-mcm7 containing 3,5,7 Ins mutant displayed reduced stability during the purification and weak binding to ORC/Cdc6. However, other mutants containing *Ins-mcm7* including 3,6,7 Ins and 4,5,7 Ins purified normal and allowed efficient OCM formation. This suggests that the structural context has an influence on complex stability—the specific mutant combination is more important than the influence of the single mutation.

Moreover, the analysis of the mutants highlighted four different categories of mutants (Figure 2C). The mutants in category 4 were capable of some salt-stable MCM2-7 loading, explaining why these mutants displayed no dominant lethality. On the other hand, the category 1–3 mutants were not able to load salt-stable MCM2-7 onto DNA, but had various *in vivo* and *in vitro* deficiencies. The category 1 mutants, in particular, displayed several defects including reduced hexamer stability (3,5,7 Ins), weak association with ORC/Cdc6 (3,5,7 Ins) and poor phosphorylation by DDK (2,5,7 Ins) and were causing strong dominant lethality. The category 2 mutants were capable of OCM formation (4,5,7 Ins and 5,6,7 Ins), but showed reduced pre-RC–induced ATPase activity (5,6,7 Ins) and caused dominant lethality on overexpression. Finally, the category 3 mutants and in particular 2,5,6 Ins was functional for OCM formation, displayed normal pre-RC–induced ATPase activity and was capable of MCM2-7 dimerization, but failed to form a stable double hexamer. It is interesting to note that the category 3 mutants (2,5,6 Ins and 3,6,7 Ins) showed near-normal growth on overexpression, but revealed dominant lethality on addition of HU. Based on our *in vitro* analysis it is likely that overexpression of these mutants causes a MCM2-7 loading defect *in vivo*. However, this effect is not sufficient to completely block DNA replication, as MCM2-7 loading is a highly redundant process, and hence we observed cell growth. Interestingly, reduced MCM2-7 loading in the human or *Xenopus* system renders cells sensitive to HU, as these cells lack sufficient MCM2-7 complexes for reactivation after a terminal fork arrest. It is tempting to speculate that reduced MCM2-7 loading due to the Ins mutation rendered the cells sensitive to HU and fork arrest.

## A model for ORC/Cdc6-mediated MCM2-7 dimerization and helicase loading

**Figure 7.**
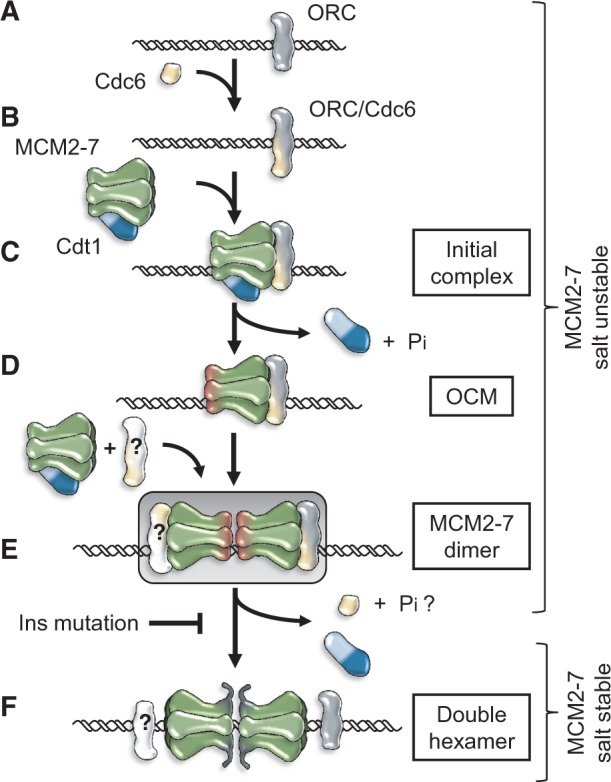
A model for ORC/Cdc6 mediated MCM2-7 dimerization and helicase loading. A model of the MCM2-7 loading process is shown. (**A**) ORC is bound to DNA. (**B**) Cdc6 associates with ORC. (**C**) Cdt1/MCM2-7 associates with ORC/Cdc6. (**D**) ATP hydrolysis leads to Cdt1 release and OCM formation. (**E**) OCM formation results in a structural change in MCM2-7 to generate a dimerization competent state of the MCM2-7 helicase and recruitment of a second MCM2-7 complex. The recruitment of the second complex may require another ORC/Cdc6 complex. (**F**) The MCM2-7 loading finishes with the formation of a stable double-hexamer encircling DNA, which leads to exposure of the Mcm2, 4 and 6 N-termini. ORC ATP hydrolysis is required for repetitive MCM2-7 loading.

Based on the coordinated and distinct structural changes in MCM2-7, which are promoted by ORC and its cofactors Cdc6 and Cdt1, we propose a model that explains how the MCM2-7 double-hexamer loading onto DNA occurs (Figure 7). ORC binds in an ATP-dependent way to the replication origin (25) (Figure 7A). In G1 phase, Cdc6 is recruited to ORC in an ATP-dependent fashion (9,26–29) (Figure 7B). Cdt1 forms a complex with MCM2-7 and this interaction is required for the initial recruitment of MCM2-7 to ORC/Cdc6 (3,4,12,14,22). Initially only one MCM2-7 hexamer is recruited to ORC/Cdc6 (15). In the absence of Cdc6 ATP hydrolysis, Cdt1 is greatly stabilized in this ‘initial’ ORC/Cdc6/Cdt1/MCM2-7 complex (31) (Figure 7C). Rapid ATP hydrolysis results in Cdt1 release and formation of an OCM complex (12) (Figure 7D). The OCM complex, but not the ‘initial’ complex, is competent for MCM2-7 dimerization (Figure 7E). Currently, it is not clear if one or two ORC/Cdc6 complexes participate in MCM2-7 dimerization or when DNA loading occurs. After initial dimerization, several important changes occur: the MCM2-7 N-termini are exposed, rendering the complex competent for DDK-mediated activation in S-phase, the stable MCM2-7 double-hexamer forms around ds-DNA, ORC and Cdc6 are released, and finally Orc1 ATP hydrolysis occurs to allow another round of MCM2-7 loading (Figure 7F).

## SUPPLEMENTARY DATA

Supplementary Data are available at NAR Online.

## FUNDING

Funding for open access charge: Imperial College London.

*Conflict of interest statement*. None declared.

## Supplementary Material

Supplementary Data
