# Supplementary material for: The ORC/Cdc6/MCM2-7 complex facilitates MCM2-7 dimerization during prereplicative complex formation
Source: Nucleic Acids Res. 2013 Nov 14;42(4):2257–69. doi: 10.1093/nar/gkt1148 (PMC3936773; doi:10.1093/nar/gkt1148)
Supplement: Supplementary Data [file supp_42_4_2257__index.html]

The ORC/Cdc6/MCM2-7 complex facilitates MCM2-7 dimerization during prereplicative complex formation — The ORC/Cdc6/MCM2-7 complex facilitates MCM2-7 dimerization during prereplicative complex formation — Supplementary Data 

# The ORC/Cdc6/MCM2-7 complex facilitates MCM2-7 dimerization during prereplicative complex formation

## Supplementary Data

files

**Files in this Data Supplement:**

- Supplementary Data - pdf file
