# Supplementary material for: The ORC/Cdc6/MCM2-7 complex facilitates MCM2-7 dimerization during prereplicative complex formation
Source: Nucleic Acids Res. 2013 Nov 14;42(4):2257–69. doi: 10.1093/nar/gkt1148 (PMC3936773; doi:10.1093/nar/gkt1148)
Supplement: Supplementary Data [file supp_gkt1148_nar-00301-h-2013-File009.pdf]

SUPPLEMENTARY DATA

The ORC/Cdc6/MCM2-7 complex facilitates MCM2-7 dimerization during pre-replicative complex formation

Cecile Evrin, Alejandra Fernández-Cid, Alberto Riera, Juergen Zech, Pippa Clarke, M. Carmen Herrera, Silvia Tognetti, Rudi Lurz, and Christian Speck

SUPPLEMENTARY FIGURES

Supplementary Figure S1 (related to main Figure 1). The influence of HU on dominant lethality of Ins MCM2-7 mutants

Supplementary Figure S2 (related to main Figure 3). Several Ins MCM2-7 mutants fractionate by gel-filtration as a hexamer

Supplementary Figure S3 (related to main Figure 3). Several Ins MCM2-7 mutants release Cdt1 efficiently in the presence of ATP

Supplementary Figure S4 (related to main Figure 5). wt MCM2-7 and Ins MCM2-7 mutants are getting phosphorylated by DDK with equal efficiency

Supplementary Figure S5 (related to main Figure 5). Quantification of loaded MCM2-7 after DDK phosphorylation

Supplementary Figure S6 (related to main Figure 6). 2,5,6 Ins MCM2-7 forms a dimer, but does not get loaded as a double-hexamer onto DNA in the presence of wt MCM2-7

SUPPLEMENTARY MATERIAL AND METHODS

SUPPLEMENTARY TABLES

Supplementary Table S1 (related to Material and Methods). Primers used to insert GGSGSG sequence in MCM2-7 subunits

Supplementary Table S2 (related to related to Material and Methods): AS499 based yeast strains used in this study

Supplementary Table S3 (related to related to Material and Methods): Plasmids used in this study

SUPPLEMENTARY REFERENCES

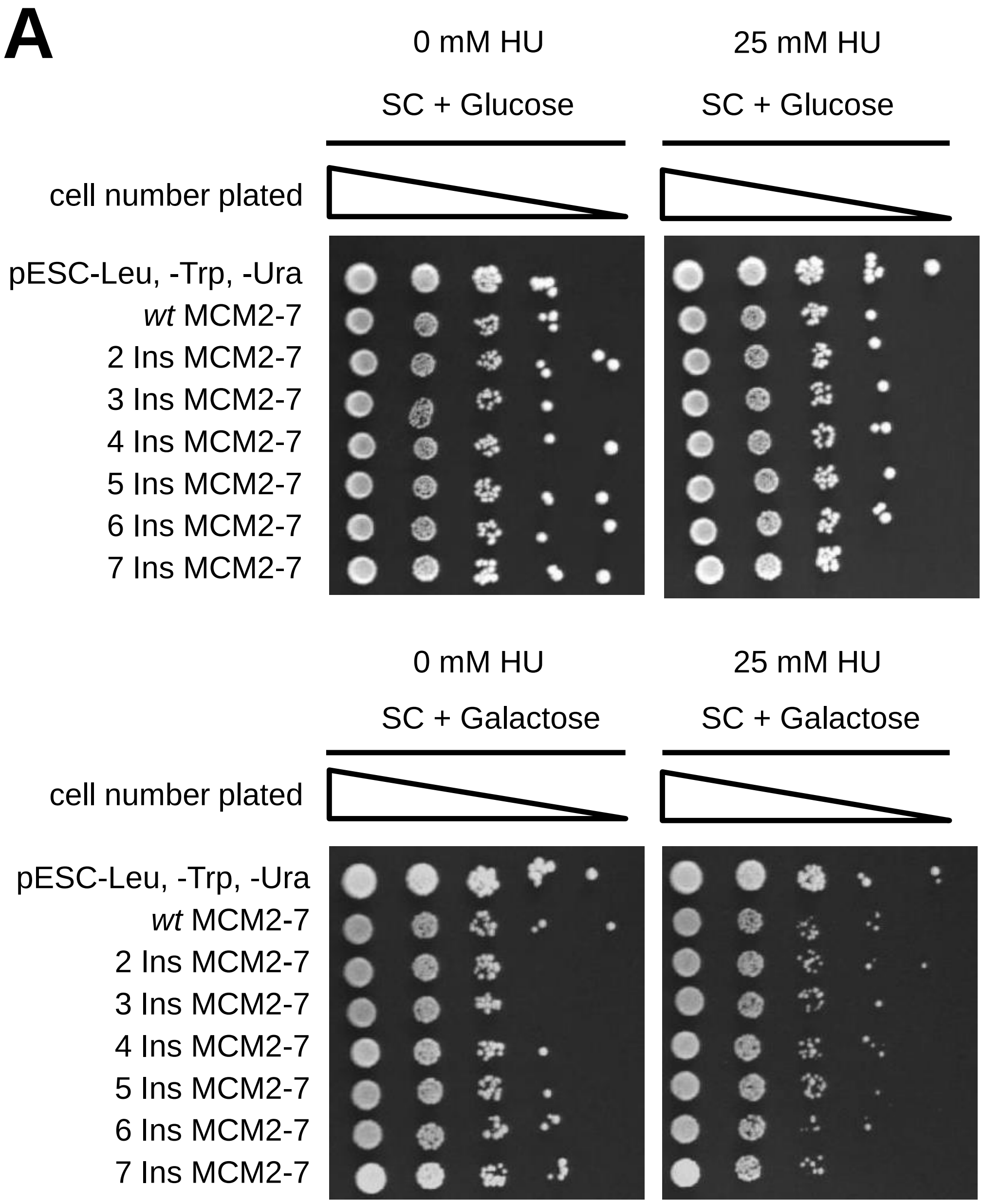
A
0 mM HU
SC + Glucose
25 mM HU
SC + Glucose
cell number plated
pESC-Leu, -Trp, -Ura
wt MCM2-7
2 Ins MCM2-7
3 Ins MCM2-7
4 Ins MCM2-7
5 Ins MCM2-7
6 Ins MCM2-7
7 Ins MCM2-7
0 mM HU
SC + Galactose
25 mM HU
SC + Galactose
cell number plated
pESC-Leu, -Trp, -Ura
wt MCM2-7
2 Ins MCM2-7
3 Ins MCM2-7
4 Ins MCM2-7
5 Ins MCM2-7
6 Ins MCM2-7
7 Ins MCM2-7

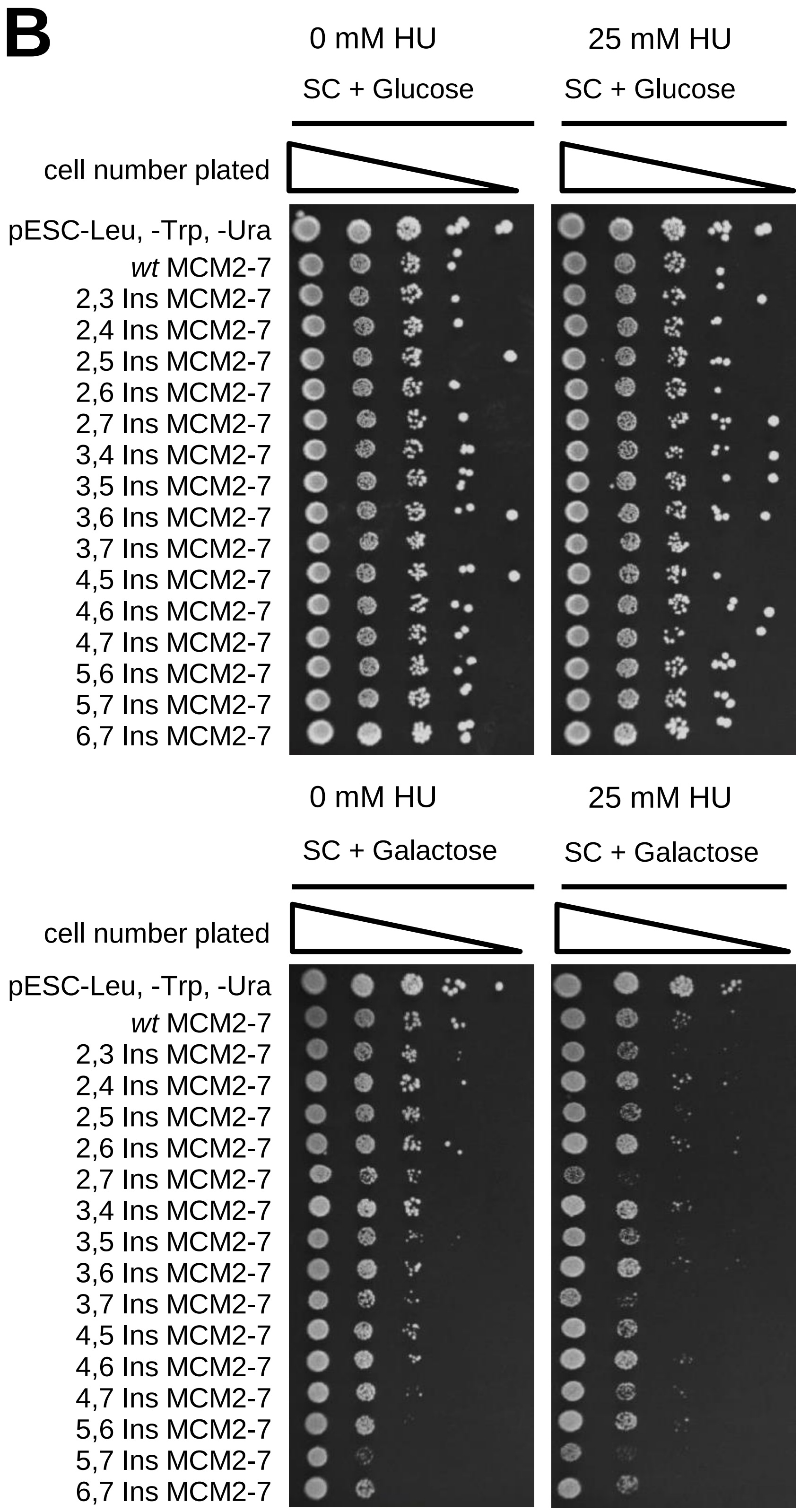
B
0 mM HU
SC + Glucose
25 mM HU
SC + Glucose
cell number plated
pESC-Leu, -Trp, -Ura
wt MCM2-7
2,3 Ins MCM2-7
2,4 Ins MCM2-7
2,5 Ins MCM2-7
2,6 Ins MCM2-7
2,7 Ins MCM2-7
3,4 Ins MCM2-7
3,5 Ins MCM2-7
3,6 Ins MCM2-7
3,7 Ins MCM2-7
4,5 Ins MCM2-7
4,6 Ins MCM2-7
4,7 Ins MCM2-7
5,6 Ins MCM2-7
5,7 Ins MCM2-7
6,7 Ins MCM2-7
0 mM HU
SC + Galactose
25 mM HU
SC + Galactose
cell number plated
pESC-Leu, -Trp, -Ura
wt MCM2-7
2,3 Ins MCM2-7
2,4 Ins MCM2-7
2,5 Ins MCM2-7
2,6 Ins MCM2-7
2,7 Ins MCM2-7
3,4 Ins MCM2-7
3,5 Ins MCM2-7
3,6 Ins MCM2-7
3,7 Ins MCM2-7
4,5 Ins MCM2-7
4,6 Ins MCM2-7
4,7 Ins MCM2-7
5,6 Ins MCM2-7
5,7 Ins MCM2-7
6,7 Ins MCM2-7

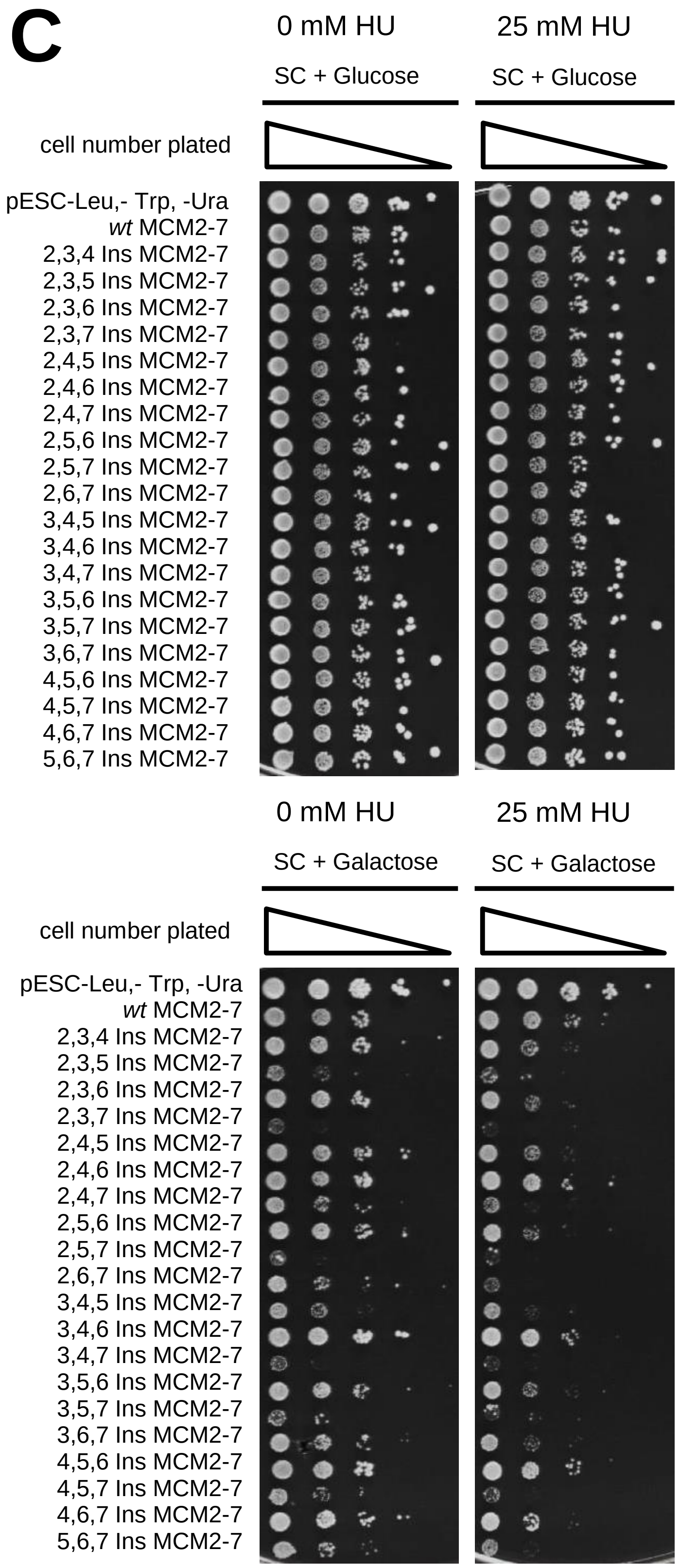
C
0 mM HU
25 mM HU
SC + Glucose
SC + Glucose
cell number plated
pESC-Leu,- Trp, -Ura
wt MCM2-7
2,3,4 Ins MCM2-7
2,3,5 Ins MCM2-7
2,3,6 Ins MCM2-7
2,3,7 Ins MCM2-7
2,4,5 Ins MCM2-7
2,4,6 Ins MCM2-7
2,4,7 Ins MCM2-7
2,5,6 Ins MCM2-7
2,5,7 Ins MCM2-7
2,6,7 Ins MCM2-7
3,4,5 Ins MCM2-7
3,4,6 Ins MCM2-7
3,4,7 Ins MCM2-7
3,5,6 Ins MCM2-7
3,5,7 Ins MCM2-7
3,6,7 Ins MCM2-7
4,5,6 Ins MCM2-7
4,5,7 Ins MCM2-7
4,6,7 Ins MCM2-7
5,6,7 Ins MCM2-7
0 mM HU
25 mM HU
SC + Galactose
SC + Galactose
cell number plated
pESC-Leu,- Trp, -Ura
wt MCM2-7
2,3,4 Ins MCM2-7
2,3,5 Ins MCM2-7
2,3,6 Ins MCM2-7
2,3,7 Ins MCM2-7
2,4,5 Ins MCM2-7
2,4,6 Ins MCM2-7
2,4,7 Ins MCM2-7
2,5,6 Ins MCM2-7
2,5,7 Ins MCM2-7
2,6,7 Ins MCM2-7
3,4,5 Ins MCM2-7
3,4,6 Ins MCM2-7
3,4,7 Ins MCM2-7
3,5,6 Ins MCM2-7
3,5,7 Ins MCM2-7
3,6,7 Ins MCM2-7
4,5,6 Ins MCM2-7
4,5,7 Ins MCM2-7
4,6,7 Ins MCM2-7
5,6,7 Ins MCM2-7

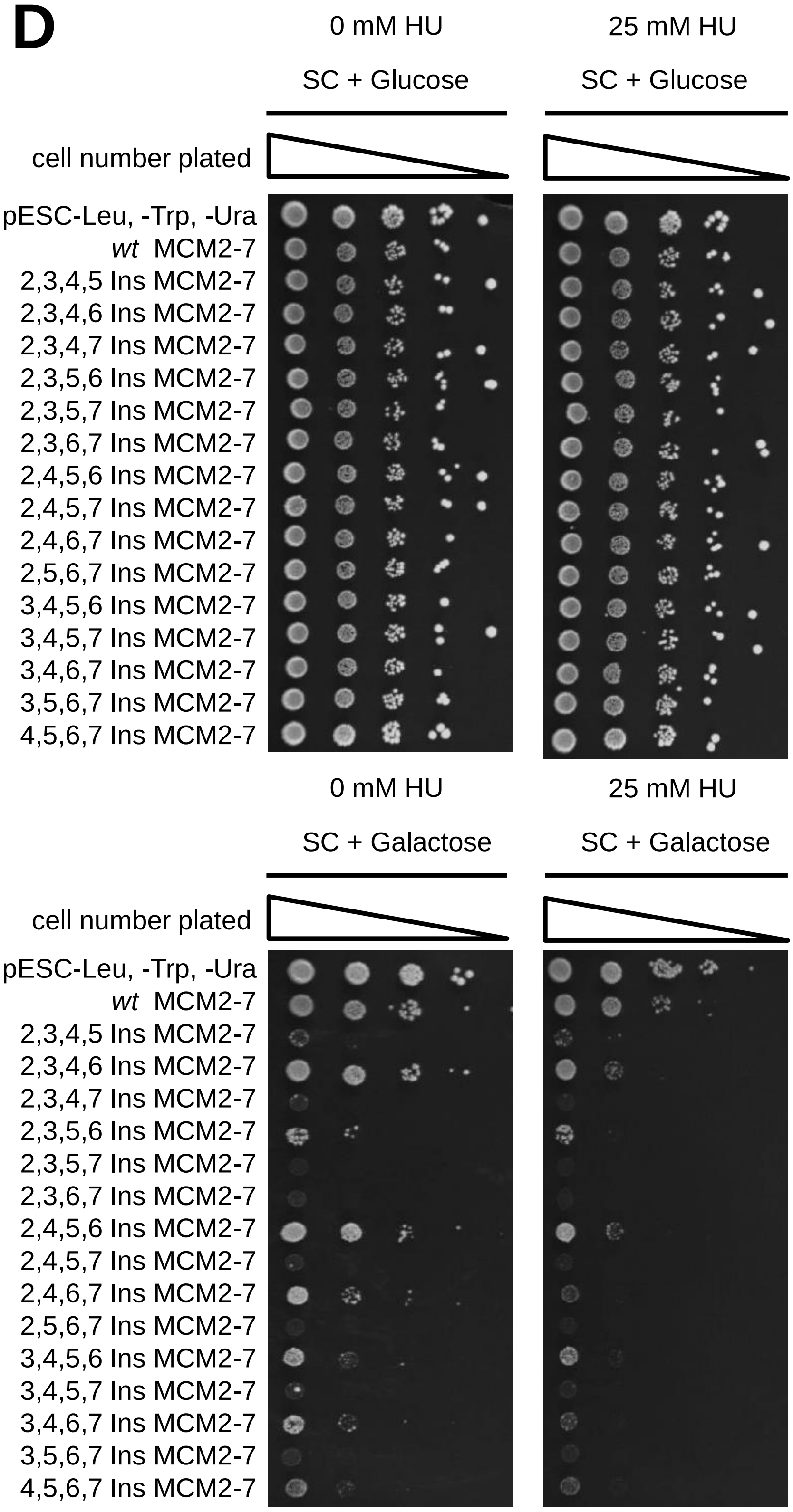
D
0 mM HU
SC + Glucose
25 mM HU
SC + Glucose
cell number plated
pESC-Leu, -Trp, -Ura
wt MCM2-7
2,3,4,5 Ins MCM2-7
2,3,4,6 Ins MCM2-7
2,3,4,7 Ins MCM2-7
2,3,5,6 Ins MCM2-7
2,3,5,7 Ins MCM2-7
2,3,6,7 Ins MCM2-7
2,4,5,6 Ins MCM2-7
2,4,5,7 Ins MCM2-7
2,4,6,7 Ins MCM2-7
2,5,6,7 Ins MCM2-7
3,4,5,6 Ins MCM2-7
3,4,5,7 Ins MCM2-7
3,4,6,7 Ins MCM2-7
3,5,6,7 Ins MCM2-7
4,5,6,7 Ins MCM2-7
0 mM HU
SC + Galactose
25 mM HU
SC + Galactose
cell number plated
pESC-Leu, -Trp, -Ura
wt MCM2-7
2,3,4,5 Ins MCM2-7
2,3,4,6 Ins MCM2-7
2,3,4,7 Ins MCM2-7
2,3,5,6 Ins MCM2-7
2,3,5,7 Ins MCM2-7
2,3,6,7 Ins MCM2-7
2,4,5,6 Ins MCM2-7
2,4,5,7 Ins MCM2-7
2,4,6,7 Ins MCM2-7
2,5,6,7 Ins MCM2-7
3,4,5,6 Ins MCM2-7
3,4,5,7 Ins MCM2-7
3,4,6,7 Ins MCM2-7
3,5,6,7 Ins MCM2-7
4,5,6,7 Ins MCM2-7

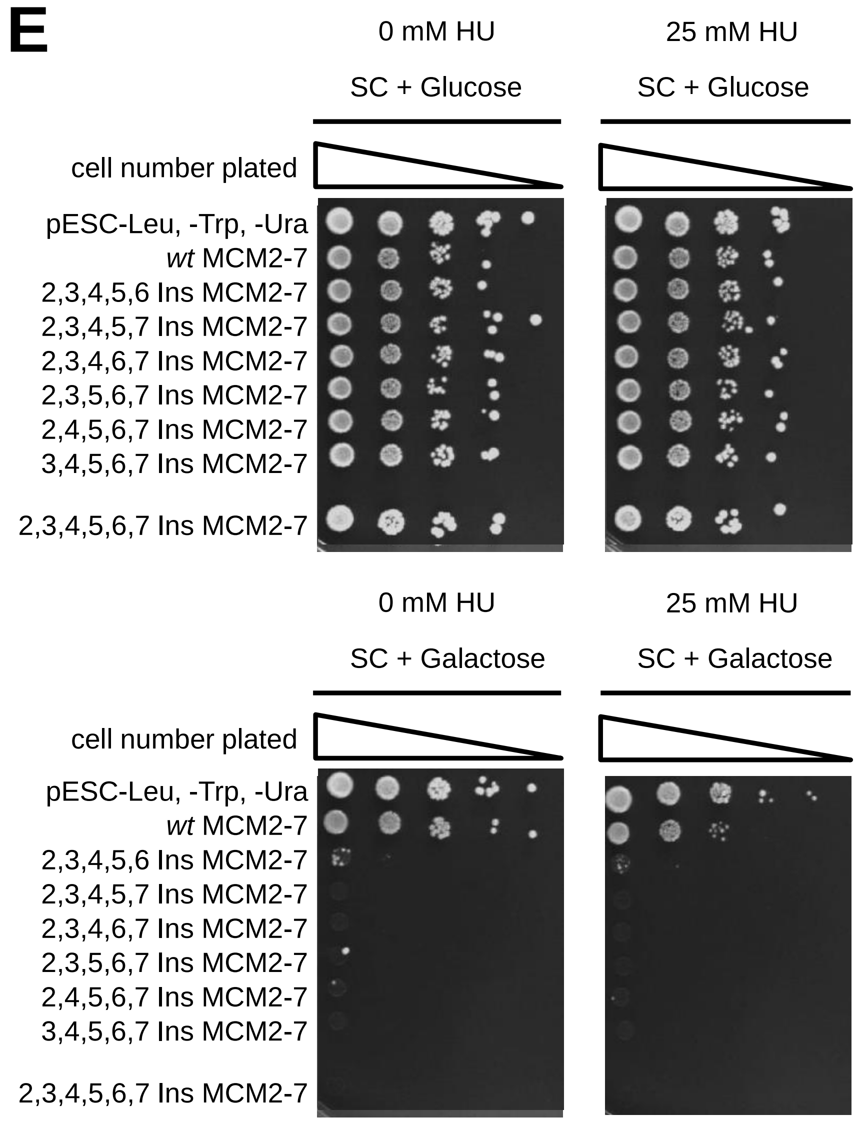

Supplementary Figure S1 (related to main Figure 1). The influence of HU on dominant lethality of Ins MCM2-7 mutants

Individual (A), double (B), triple (C), quadruple (D), quintuple and sextuple (E) Ins mutants were overexpressed (galactose) or not expressed (glucose) from 2-micron plasmids to test for dominant lethal effects in the presence or absence of 25 mM HU. The figures in absence of HU are duplicated from main figure 1. All tested strains contained in addition to the mutants the complementing *wt* Mcm genes so that always all six MCM2-7 genes got co-expressed.

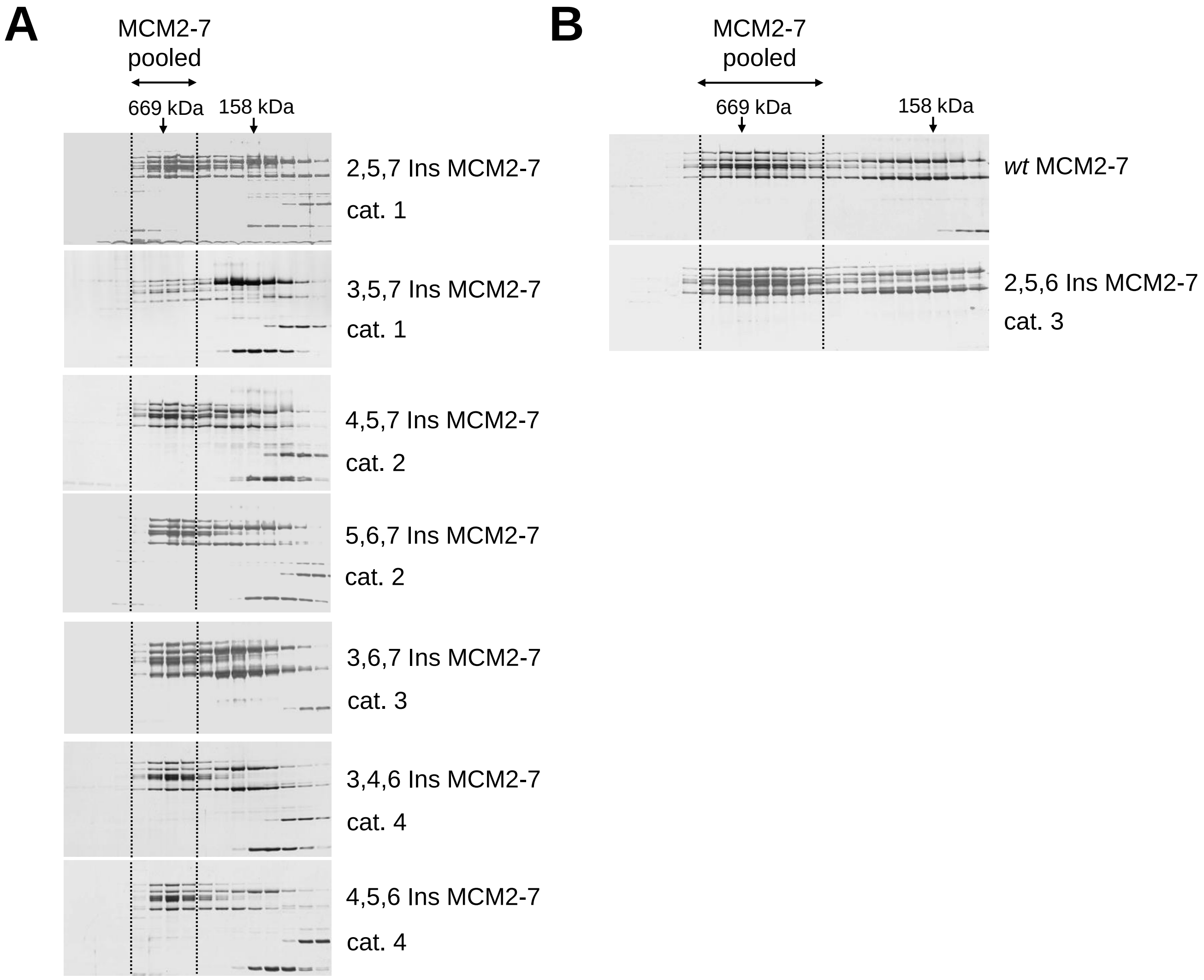

Supplementary Figure S2 (related to main Figure 3). Several Ins MCM2-7 mutants fractionate by gel-filtration as a hexamer

The stability of the Ins mutant complexes was evaluated by gel-filtration on Superose 6 or Superdex 200 columns. The proteins were loaded on the column after HA-affinity purification. (A) 2,5,7 Ins, 3,5,7 Ins, 4,5,7 Ins, 5,6,7 Ins, 3,6,7 Ins, 3,4,6 Ins and 4,5,6 Ins triple mutants were fractionated (Superose 6). (B) wt MCM2-7 and 2,5,6 Ins were fractionated (Superdex 200).

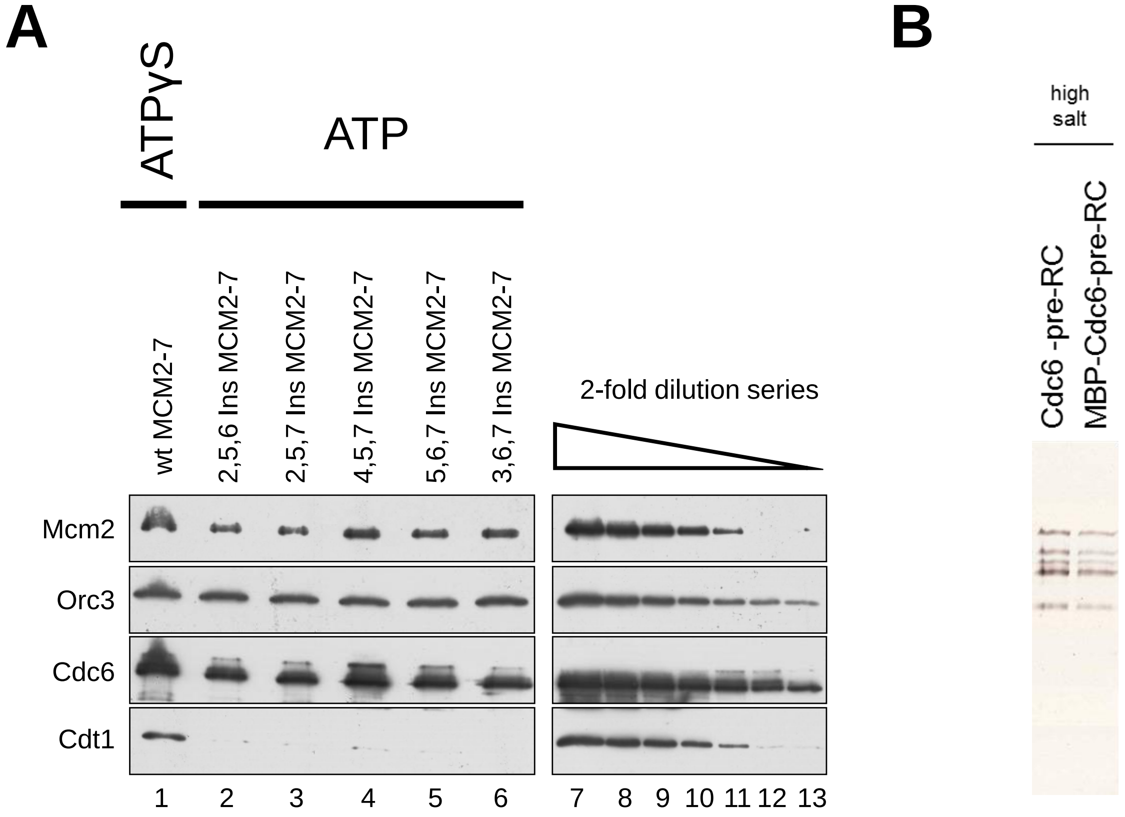

Supplementary Figure S3 (related to main Figure 3). Several Ins MCM2-7 mutants release Cdt1 efficiently in the presence of ATP.

(A) Pre-RC complexes with *wt* MCM2-7 or five distinct Ins MCM2-7 mutants were formed in the presence of ATPγS (lanes 1) or ATP (lanes 2-7) and the resulting DNA-bound complexes were analyzed for the presence of ORC, MCM2-7, MBP-Cdc6 and Cdt1 using anti-Orc3, anti-Mcm2, anti-MBP (Cdc6) and anti-Cdt1 antibodies. A dilution series of these proteins is shown with 40%, 20%, 10%, 5%, 2.5%, 1.25% and 0.625% of the input of one pre-RC assembly reaction (lanes 7-13). (B) A pre-RC assay was performed with Cdc6 and MBP-Cdc6 followed by a high salt wash, showing only high-salt resistant loaded MCM2-7. This is a control for A, showing that MBP-Cdc6 loads MCM2-7 with similar efficiency as untagged Cdc6.

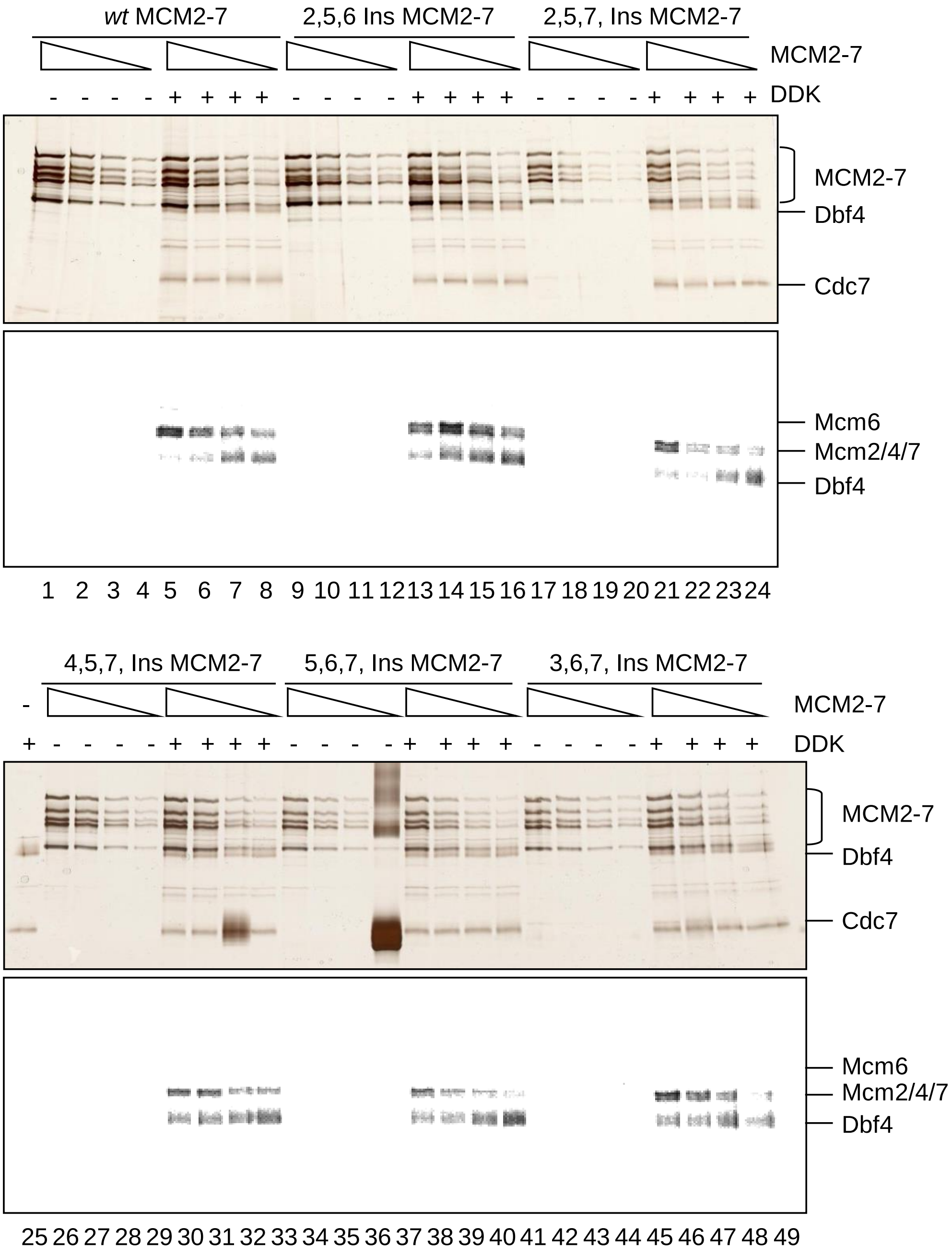

Supplementary Figure S4 (related to main Figure 5). *wt* MCM2-7 and Ins MCM2-7 mutants are getting phosphorylated by DDK with equal efficiency

Soluble hexameric MCM2-7 dilution were incubated with or without DDK. *wt* MCM2-7 control is shown in lanes 1-8. A DDK control lane is shown in lane 25. The upper panel shows silver stained gels and the lower panel the autoradiogram of the same gel. 40, 20, 10 or 5 nM wt MCM2-7 (lanes 1-8), 2,5,6 Ins MCM2-7 (lanes 9-16), 2,5,7 Ins MCM2-7 (lanes 17-24), 4,5,7 Ins MCM2-7 (lanes 26-33), 5,6,7 Ins MCM2-7 (lanes 34-41) and 3,6,7 Ins MCM2-7 (lanes

42-49) were incubated for 15 min at 27°C either in the absence (lanes 1-4, 9-12, 17-20, 26-29, 34-37 and 42-45) or in the presence (lanes 5-8, 13-16, 21-24, 30-33, 38-41 and 46-49) of 40 nM DDK and γ32P-ATP. Lane 25 contains 40 nM DDK.

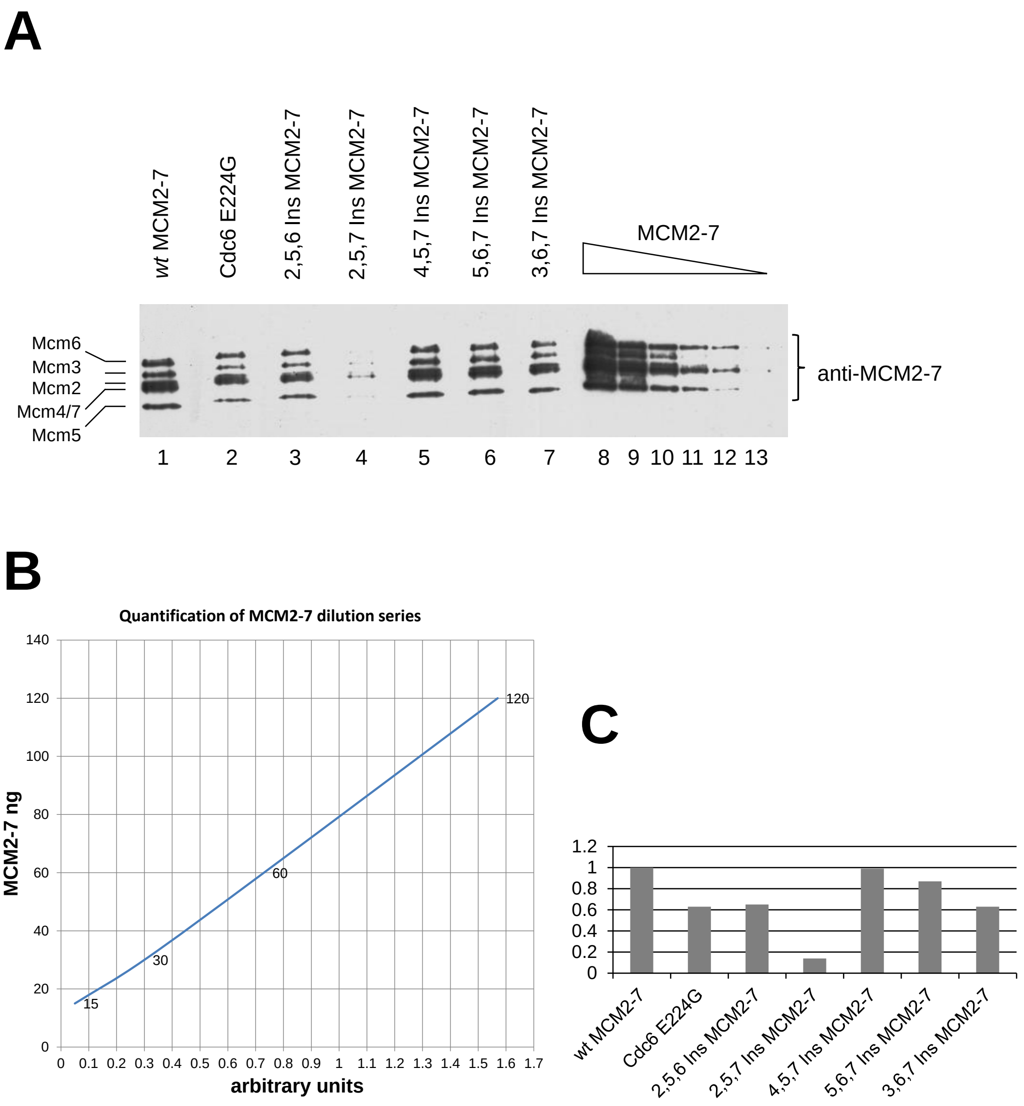

Supplementary Figure S5 (related to main Figure 5). Quantification of associated MCM2-7 after DDK phosphorylation

(A) The experiment was performed as in figure 5 and serves as a control for the same experiment. In lanes 8-13 an MCM2-7 dilution series is shown with 480 ng, 240 ng, 120 ng, 60 ng, 30 ng and 15 ng of MCM2-7. An anti-MCM2-7 antibody was used to quantify the amount of MCM2-7 after DDK phosphorylation. The pre-RC assembly was performed with 6 nM pUC19-ARS1, 40 nM ORC, 80 nM Cdc6 or Cdc6 E224G, 40 nM Cdt1 and 40 nM *wt* MCM2-7 or 40 nM Ins MCM2-7 mutants. Samples were incubated with 40 nM DDK.(B) Quantification of the MCM2-7 dilution series (15-120 ng). (C) Quantification of A.

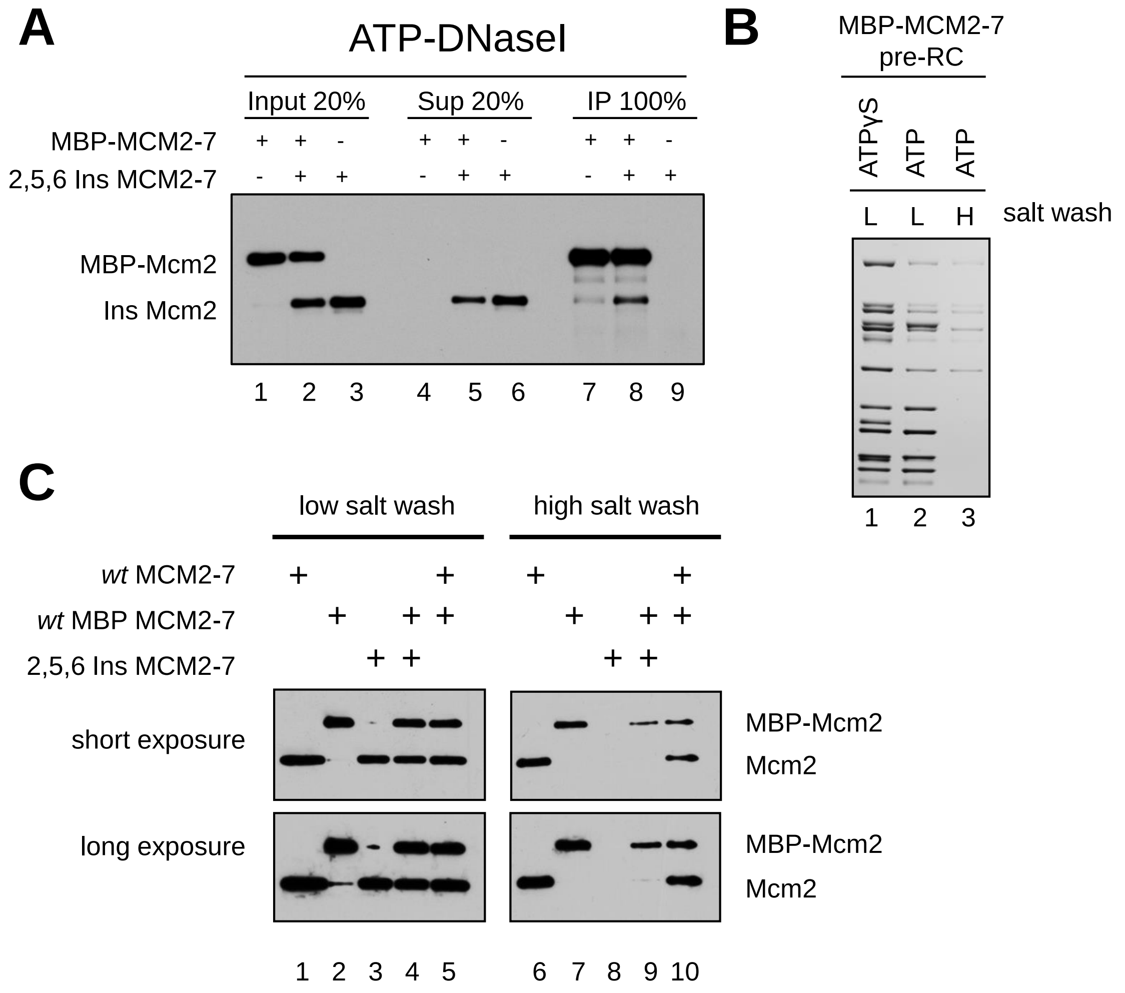

Supplementary Figure S6 (related to main Figure 6). 2,5,6 Ins MCM2-7 forms a dimer, but does not get loaded as a double-hexamer onto DNA in the presence of *wt* MCM2-7

(A) A pre-RC assay was performed with MBP-MCM2-7 with ATPγS and ATP followed by a low or high salt wash. We observed that MBP-MCM2-7 was loaded into a high salt resistant complex. (B) Pre-RC assays were assembled in the presence of ATP. When tagged and untagged MCM2-7 were used equimolar amounts of each complex were combined in pre-RC reactions. Complexes were released from DNA via DNase I and DNA bound complexes were immunoprecipitated (IP) and analysed by western blotting together with input and supernatant (Sup) using anti-Mcm2 antibody. (C) The efficiency of 2,5,6 Ins MCM2-7 in loading was evaluated by using a reconstituted pre-RC system employing purified ORC (40 nM), Cdc6 (80 nM), Cdt1 (40 nM), wt MCM2-7 (40 nM), wt MBP MCM2-7 (40 nM), 2,5,6 Ins MCM2-7 (40 nM) and origin containing DNA (6 nM). The reactions were assembled and

washed with low salt (lanes 1-5) or high salt (lanes 6-10) and analysed by western-blotting using an anti-Mcm2 antibody. The high salt sample indicates the amount of loaded MCM2-7. A short and long exposure of the experiment is shown. Note that 2,5,6 Ins MCM2-7 is associating well in the presence of low salt, but becomes destabilized in the presence of high salt (compare lanes 3 and 4 with 8 and 9).

SUPPLEMENTARY MATERIAL AND METHODS

Cloning of Ins mutants

A six-amino-acid peptide (GGSGSG) was inserted right after a conserved cysteine (see Supplementary Table S1) using a QuickChange Lighting site-directed mutagenesis kit (MCM2,3,4,5,6) or a QuickChange II XL site-directed mutagenesis kit (MCM7) from Stratagene with the forward and reverse primers listed in Supplementary Table S1. All clones were sequenced to confirm the presence of the insertion. Ins mcm5 subunit contains an additional mutation I248N. The plasmids are listed in

Purification of pre-RC proteins

The ORC (Klemm et al, 1997), Cdc6 (Speck et al, 2005), Cdct1 and MCM2-7 (Evrin et al, 2009) and MBP-Mcm2-7 (Evrin et al, 2013) were prepared as previously described. The Ins MCM2-7 mutant and MBP-MCM2-7 proteins were expressed and purified as the wild-type MCM2-7 complex.

Cloning, expression and purification of DDK

MBP-Dbf4 and Cdc7 were cloned into pESC-Trp yeast epitope tagging vector (Stratagene). The resulting plasmid (pCS313) was transformed in strain AS499 (Evrin et al, 2009). Cells were grown in SCGL medium overnight then supplemented with an equal volume of rich medium the following morning. After 3 h, protein expression was induced by addition of 2% galactose for 3 h. Cells were lysed in a freezer mill. The resulting extract was incubated with amylose resin (NEB) pre-equilibrated with buffer C [50 mM Hepes-KOH pH 7.5, 400 mM NaCl, 1 mM EDTA, 1 mM EGTA, 10% glycerol, 0.02% NP40, 1 mM DTT] and supplemented with proteases and phosphatases inhibitors for 2 h at 4°C. The protein was eluted by cleaving the MBP tag via addition of PreScission protease for 2 h at 4°C. The eluate was incubated with Glutathione agarose for 2 h to remove the PreScission protease. The GST flow-through was collected, concentrated and stored at -80°C.

In vitro kinase assay - phosphorylation of pre-RC proteins in solution

5 nM to 40 nM of purified proteins were incubated with 40 nM DDK in a final volume of 10 µl buffer P [50 mM Hepes-KOH pH 7.5, 100 mM KGlu, 10 mM MgAc, 50 µM ZnAc, 0.1 mM ATP, 5 mM DTT, 0.1% Triton X-100, and 5% Glycerol, 100 µCi/ml γ-^{32}P ATP] for 15 min at 27°C. Proteins were separated on a 7.5% SDS-polyacrylamide gel and silver stained. The incorporation of ^{32}P-phosphate was detected by autoradiography.

In vitro kinase assay - phosphorylation of pre-RC proteins on DNA beads

The pre-RCs were formed as described above with minor modifications. First, Lambda phosphatase was omitted from the reaction. Second, following the wash with buffer A and prior to the DNaseI elution the beads were re-suspended in buffer P containing 40 nM DDK and incubated at 27°C for 15 min. After an additional wash with buffer P without the radioactive nucleotide, the DNA was digested

by DNaseI as before. Proteins were separated on a 7.5% SDS-polyacrylamide gel and silver stained or analysed by western blot. The incorporation of ^{32}P-phosphate was detected by autoradiography of the silver stained gel and quantified with Multi Gauge (FUJI). The MCM2-7 protein amounts were quantified based on a dilution series analyzed by western blotting. The quantitative densiometric analysis of the western-blot was performed on digitized images of film using Multi Gauge (FUJI). The obtained values for the dilution series are shown in Supplementary Figure S4B. The quantified amount of MCM2-7 on DNA is shown in Supplementary Figure S4C and these data were used to compensate for differences in MCM2-7 binding in Figure 5B. Consequently we calculated the DDK dependent phosphorylation of wt MCM2-7 and the Ins mutants (Figure 5D), which were corrected for the MCM2-7 protein amounts.

ATPase assay

The ATPase assay was performed as described (Speck & Stillman, 2007) with minor modifications. We used a 150 bp DNA fragment containing the ARS1 sequence that was amplified with primer ARS150 FWD (CAAAATAGCAAATTTCGTCAAAAATGC) and primer ARS1 150 REV (TTTACATCTTGTTATTTTACAGATTTTATGTTTAGATC) from the plasmid pCS372 - (pUC19 ARS1). 2.5 pmol of wt or mutant ORC, Cdc6, Cdt1 and MCM2-7 were incubated for 30 min on ice in 12 µl of ATPase buffer [25 mM Hepes, pH 7.6, 100 mM KGlu, 5mM MgAc, 1mM DTT, 1 mM EDTA, 1 mM EGTA, 0.1% (v/v) Triton X-100, 10% glycerol] containing 2.5 pmol of DNA (when indicated) and 1 mM ATP. After the incubation, 5 µCi of [α-32P] ATP (3000 Ci/mmol) was added and the reaction was started by moving the tubes from ice into a 25°C water bath. 2 µl aliquots were collected at 15, 30, 45, and 60 min and stopped with 0.5 µl of stop solution (2% SDS, 50 mM EDTA). 1 µl of the samples were consequently spotted on TLC plates and developed. Error bars represent the standard deviation from at least three independent experiments.

Yeast growth assay on plates

Yeast strains used are listed in Supplementary Table S2. The strain YC41 was transformed with the corresponding plasmids listed in Supplementary Table S3. All strains were grown overnight in selective medium at 30°C. A ten-fold serial dilution starting from 10^7 cells were spotted on selective plates in the presence of either 2% glucose or galactose. Plates were incubated at 30°C for 4 days.

Yeast growth conditions and Flow cytometry analysis

Cells were grown to an OD_{600} = 0.5 at 30°C in SC containing 2% raffinose before adding nocodazole at a concentration of 5 ng/µl to arrest cells at G2/M. 2% galactose was added to induce *wt* MCM or Ins MCM2-7 mutants expression for 2 h. Cells were then released into fresh YP containing 2% raffinose and 2% galactose. Aliquots of the cells were taken throughout the experiment and their progression into the cell cycle was monitored by flow cytometry. Cells were fixed in 70% ethanol, sonicated for 15 s at 30% amplitude, treated with 0.25 mg/ml RNaseA at 50°C for 1h, and then stained with 16 µg/ml of propidium iodide. Samples were analyzed on LSRII instrument and FACS profiles prepared with FlowJo software.

SUPPLEMENTARY TABLES

Supplementary Table S1 (related to Experimental procedures). Primers used to insert GGSGSG sequence in MCM2-7 subunits

MCM2-7 subunit	Amino acid 1	Amino acid 2	Forward primers	Reverse primers
2	Cys367	Lys368	5’tgactctaaagggaccttttgatttaccgctgccggatccgccgcagtttgtacagaatgag	5’ctcattctgtacaaactgcggcggatccggcagcggtaaatcaaaaggtccctttagagtca
3	Thr236	Glu237	5’ctatccaacggaggacactggcggatccggcagcggtgaaggtaacaaactaacca	5’tggttagtttgttaccttcaccgctgccggatccgccagtgtcctccgttggatag
4	Cys376	Asn378	5’taggtgtgaacgtattgattgtggcggatccggcagcggtaatgaacccaattccatgtcac	5’gtgacatggaattgggttcattaccgctgccggatccgccacaatcaatacgttcacaccta
5	Cys236	Gly237	5’gataataatatatggatcaggtccaccgctgccggatccgccacaatttttcttggtcgattcatc	5’gatgaatcgaccaagaaaaattgtggcggatccggcagcggtggacctgatccatatattattatc
6	Cys338	Glu339	5’tgtccaaaaggctctattttcaccgctgccggatccgccgcatgatgggttcggg	5’cccgaacccatcatgcggcggatccggcagcggtgaaaatagagccttttggaca
7	Cys289	Gln290	5’cgttgtcagaatgtacttccgaagaatgtggcggatccggcagcggttcccaaaatcaaacaaa	5’tttgtttgattttgggaaccgctgccggatccgccacattcttcggaagtacattctgacaacg

Supplementary Table S2 (related to Experimental procedures): AS499 based yeast strains used in this study

Strains	genotype	MCM2-7
YC41	*MATa bar1Δ leu2-3,112 ura3-52 his3-Δ200 trp1-Δ 63 ade2-1 lys2-801 pep4* pESC-LEU pESC-TRP pESC-URA	none
YC119	*MATa bar1Δ leu2-3,112 ura3-52 his3-Δ200 trp1-Δ 63 ade2-1 lys2-801 pep4* pESC-LEU-MCM2-MCM7 pESC-TRP-MCM6-MCM4 pESC-URA-HA_MCM3-MCM5	wt
YC206	*MATa bar1Δ leu2-3,112 ura3-52 his3-Δ200 trp1-Δ 63 ade2-1 lys2-801 pep4* pESC-LEU-**InsMCM2**-MCM7 pESC-TRP-MCM6-**InsMCM4** pESC-URA-HA_MCM3-**InsMCM5**	2,4,5 Ins
YC207	*MATa bar1Δ leu2-3,112 ura3-52 his3-Δ200 trp1-Δ 63 ade2-1 lys2-801 pep4* pESC-LEU-**InsMCM2**-MCM7 pESC-TRP-**InsMCM6**-MCM4 pESC-URA-HA_MCM3-**InsMCM5**	2,5,6 Ins
YC208	*MATa bar1Δ leu2-3,112 ura3-52 his3-Δ200 trp1-Δ 63 ade2-1 lys2-801 pep4* pESC-LEU-**InsMCM2**-MCM7 pESC-TRP-MCM6-MCM4 pESC-URA-HA_MCM3-MCM5	2 Ins
YC209	*MATa bar1Δ leu2-3,112 ura3-52 his3-Δ200 trp1-Δ 63 ade2-1 lys2-801 pep4* pESC-LEU-MCM2-MCM7 pESC-TRP-MCM6-MCM4 pESC-URA-**InsHA-MCM3**-MCM5	3 Ins
YC210	*MATa bar1Δ leu2-3,112 ura3-52 his3-Δ200 trp1-Δ 63 ade2-1 lys2-801 pep4* pESC-LEU-MCM2-MCM7 pESC-TRP-MCM6-MCM4 pESC-URA-HA_MCM3-**InsMCM5**	5 Ins
YC211	*MATa bar1Δ leu2-3,112 ura3-52 his3-Δ200 trp1-Δ 63 ade2-1 lys2-801 pep4* pESC-LEU-MCM2-MCM7 pESC-TRP-**InsMCM6**-MCM4 pESC-URA-HA_MCM3-MCM5	6 Ins
YC212	*MATa bar1Δ leu2-3,112 ura3-52 his3-Δ200 trp1-Δ 63 ade2-1 lys2-801 pep4* pESC-LEU-**InsMCM2**-MCM7 pESC-TRP-MCM6-MCM4 pESC-URA-**InsHA_MCM3**-MCM5	2,3 Ins
YC213	*MATa bar1Δ leu2-3,112 ura3-52 his3-Δ200 trp1-Δ 63 ade2-1 lys2-801 pep4* pESC-LEU-**InsMCM2**-MCM7 pESC-TRP-MCM6-MCM4 pESC-URA-HA_MCM3-**InsMCM5**	2,5 Ins
YC214	*MATa bar1Δ leu2-3,112 ura3-52 his3-Δ200 trp1-Δ 63 ade2-1 lys2-801 pep4* pESC-LEU-**InsMCM2**-MCM7 pESC-TRP-**InsMCM6**-MCM4 pESC-URA-HA_MCM3-MCM5	2,6 Ins
YC215	*MATa bar1Δ leu2-3,112 ura3-52 his3-Δ200 trp1-Δ 63 ade2-1 lys2-801 pep4* pESC-LEU-MCM2-MCM7 pESC-TRP-**InsMCM6**-MCM4 pESC-URA-**InsHA_MCM3**-MCM5	3,6 Ins
YC216	*MATa bar1Δ leu2-3,112 ura3-52 his3-Δ200 trp1-Δ 63 ade2-1 lys2-801 pep4* pESC-LEU-MCM2-MCM7 pESC-TRP-**InsMCM6**-MCM4 pESC-URA-HA_MCM3-**InsMCM5**	5,6 Ins
YC217	*MATa bar1Δ leu2-3,112 ura3-52 his3-Δ200 trp1-Δ 63 ade2-1 lys2-801 pep4* pESC-LEU-**InsMCM2**-MCM7 pESC-TRP-**InsMCM6**-MCM4 pESC-URA-**InsHA_MCM3**-MCM5	2,3,6 Ins
YC218	*MATa bar1Δ leu2-3,112 ura3-52 his3-Δ200 trp1-Δ 63 ade2-1 lys2-801 pep4* pESC-LEU-MCM2-MCM7 pESC-TRP-MCM6-MCM4 pESC-URA-**InsHA_MCM3**-**InsMCM5**	3,5 Ins
YC219	*MATa bar1Δ leu2-3,112 ura3-52 his3-Δ200 trp1-Δ 63 ade2-1 lys2-801 pep4* pESC-LEU-**InsMCM2**-MCM7 pESC-TRP-MCM6-MCM4 pESC-URA-**InsHA_MCM3**-**InsMCM5**	2,3,5 Ins
YC220	*MATa bar1Δ leu2-3,112 ura3-52 his3-Δ200 trp1-Δ 63 ade2-1 lys2-801 pep4* pESC-LEU-MCM2-MCM7 pESC-TRP-**InsMCM6**-MCM4 pESC-URA-**InsHA_MCM3**-**InsMCM5**	3,5,6 Ins
YC221	*MATa bar1Δ leu2-3,112 ura3-52 his3-Δ200 trp1-Δ 63 ade2-1 lys2-801 pep4* pESC-LEU-**InsMCM2**-MCM7 pESC-TRP-**InsMCM6**-MCM4 pESC-URA-**InsHA_MCM3**-**InsMCM5**	2,3,5,6 Ins
YC222	*MATa bar1Δ leu2-3,112 ura3-52 his3-Δ200 trp1-Δ 63 ade2-1 lys2-801 pep4* pESC-LEU-**InsMCM2**-MCM7 pESC-TRP-MCM6-**InsMCM4** pESC-URA-**InsHA_MCM3**-MCM5	2,3,4 Ins
YC223	*MATa bar1Δ leu2-3,112 ura3-52 his3-Δ200 trp1-Δ 63 ade2-1 lys2-801 pep4* pESC-LEU-MCM2-**InsMCM7** pESC-TRP-**InsMCM6**-MCM4 pESC-URA**6InsHA_MCM3**-MCM5	3,6,7 Ins
YC224	*MATa bar1Δ leu2-3,112 ura3-52 his3-Δ200 trp1-Δ 63 ade2-1 lys2-801 pep4* pESC-LEU-MCM2-MCM7 pESC-TRP-**InsMCM6**-**InsMCM4** pESC-URA-**InsHA_MCM3**-MCM5	3,4,6 Ins
YC225	*MATa bar1Δ leu2-3,112 ura3-52 his3-Δ200 trp1-Δ 63 ade2-1 lys2-801 pep4* pESC-LEU-MCM2-MCM7 pESC-TRP-MCM6-**InsMCM4** pESC-URA-**InsHA_MCM3**-**InsMCM5**	3,4,5 Ins
YC226	*MATa bar1Δ leu2-3,112 ura3-52 his3-Δ200 trp1-Δ 63 ade2-1 lys2-801 pep4* pESC-LEU-MCM2-**InsMCM7** pESC-TRP-MCM6-**InsMCM4** pESC-URA**-InsHA_MCM3**-MCM5	3,4,7 Ins
YC227	*MATa bar1Δ leu2-3,112 ura3-52 his3-Δ200 trp1-Δ 63 ade2-1 lys2-801 pep4* pESC-LEU-MCM2-**InsMCM7** pESC-TRP-MCM6-**InsMCM4** pESC-URA-HA_MCM3-**InsMCM5**	4,5,7 Ins
YC228	*MATa bar1Δ leu2-3,112 ura3-52 his3-Δ200 trp1-Δ 63 ade2-1 lys2-801 pep4* pESC-LEU-MCM2-**InsMCM7** pESC-TRP-**InsMCM6**-**InsMCM4** pESC-URA-HA_MCM3-MCM5	4,6,7 Ins
YC229	*MATa bar1Δ leu2-3,112 ura3-52 his3-Δ200 trp1-Δ 63 ade2-1 lys2-801 pep4* pESC-LEU-MCM2-MCM7 pESC-TRP-**InsMCM6**-**InsMCM4** pESC-URA-HA_MCM3-**InsMCM5**	4,5,6 Ins
YC230	*MATa bar1Δ leu2-3,112 ura3-52 his3-Δ200 trp1-Δ 63 ade2-1 lys2-801 pep4* pESC-LEU-MCM2-**InsMCM7** pESC-TRP-**InsMCM6**-MCM4 pESC-URA-HA_MCM3-**InsMCM5**	5,6,7 Ins
YC231	*MATa bar1Δ leu2-3,112 ura3-52 his3-Δ200 trp1-Δ 63 ade2-1 lys2-801 pep4* pESC-LEU-MCM2-**InsMCM7** pESC-TRP-MCM6-MCM4 pESC-URA-**InsHA_MCM3**-**InsMCM5**	3,5,7 Ins
YC232	*MATa bar1Δ leu2-3,112 ura3-52 his3-Δ200 trp1-Δ 63 ade2-1 lys2-801 pep4* pESC-LEU-**InsMCM2**-MCM7 pESC-TRP-MCM6-**InsMCM4** pESC-URA-**InsHA_MCM3**-**InsMCM5**	2,3,4,5 Ins
YC233	*MATa bar1Δ leu2-3,112 ura3-52 his3-Δ200 trp1-Δ 63 ade2-1 lys2-801 pep4* pESC-LEU-**InsMCM2**-MCM7 pESC-TRP-**InsMCM6**-**InsMCM4** pESC-URA-**InsHA_MCM3**-**InsMCM5**	2,3,4,5,6 Ins
YC234	*MATa bar1Δ leu2-3,112 ura3-52 his3-Δ200 trp1-Δ 63 ade2-1 lys2-801 pep4* pESC-LEU-**InsMCM2**-MCM7 pESC-TRP-**InsMCM6**-**InsMCM4** pESC-URA-HA_MCM3-MCM5	2,4,6 Ins
YC235	*MATa bar1Δ leu2-3,112 ura3-52 his3-Δ200 trp1-Δ 63 ade2-1 lys2-801 pep4* pESC-LEU-**InsMCM2**-MCM7 pESC-TRP-**InsMCM6**-**InsMCM4** pESC-URA-**InsHA_MCM3**-MCM5	2,3,4,6 Ins
YC236	*MATa bar1Δ leu2-3,112 ura3-52 his3-Δ200 trp1-Δ 63 ade2-1 lys2-801 pep4* pESC-LEU-**InsMCM2**-MCM7 pESC-TRP-**InsMCM6**-**InsMCM4** pESC-URA-HA_MCM3-**InsMCM5**	2,4,5,6 Ins
YC237	*MATa bar1Δ leu2-3,112 ura3-52 his3-Δ200 trp1-Δ 63 ade2-1 lys2-801 pep4* pESC-LEU-MCM2-MCM7 pESC-TRP-**InsMCM6**-**InsMCM4** pESC-URA-**InsHA_MCM3**-**InsMCM5**	3,4,5,6 Ins

YC238	*MATa bar1Δ leu2-3,112 ura3-52 his3-Δ200 trp1-Δ 63 ade2-1 lys2-801 pep4* pESC-LEU-MCM2-**InsMCM7** pESC-TRP-MCM6-**InsMCM4** pESC-URA-**InsHA_MCM3**-**InsMCM5**	3,4,5,7 Ins
YC239	*MATa bar1Δ leu2-3,112 ura3-52 his3-Δ200 trp1-Δ 63 ade2-1 lys2-801 pep4* pESC-LEU-MCM2-**InsMCM7** pESC-TRP-**InsMCM6**-MCM4 pESC-URA-**InsHA_MCM3**-**InsMCM5**	3,5,6,7 Ins
YC240	*MATa bar1Δ leu2-3,112 ura3-52 his3-Δ200 trp1-Δ 63 ade2-1 lys2-801 pep4* pESC-LEU-MCM2-**InsMCM7** pESC-TRP-**InsMCM6**-**InsMCM4** pESC-URA-HA_MCM3-**InsMCM5**	4,5,6,7 Ins
YC241	*MATa bar1Δ leu2-3,112 ura3-52 his3-Δ200 trp1-Δ 63 ade2-1 lys2-801 pep4* pESC-LEU-**InsMCM2**-**InsMCM7** pESC-TRP-MCM6-MCM4 pESC-URA-**InsHA_MCM3**-MCM5	2,3,7 Ins
YC242	*MATa bar1Δ leu2-3,112 ura3-52 his3-Δ200 trp1-Δ 63 ade2-1 lys2-801 pep4* pESC-LEU-**InsMCM2**-**InsMCM7** pESC-TRP-MCM6-**InsMCM4** pESC-URA-HA_MCM3-MCM5	2,4,7 Ins
YC243	*MATa bar1Δ leu2-3,112 ura3-52 his3-Δ200 trp1-Δ 63 ade2-1 lys2-801 pep4* pESC-LEU-**InsMCM2**-**InsMCM7** pESC-TRP-MCM6-MCM4 pESC-URA-HA_MCM3-**InsMCM5**	2,5,7 Ins
YC244	*MATa bar1Δ leu2-3,112 ura3-52 his3-Δ200 trp1-Δ 63 ade2-1 lys2-801 pep4* pESC-LEU-**InsMCM2**-**InsMCM7** pESC-TRP-**InsMCM6**-MCM4 pESC-URA-HA_MCM3-MCM5	2,6,7 Ins
YC245	*MATa bar1Δ leu2-3,112 ura3-52 his3-Δ200 trp1-Δ 63 ade2-1 lys2-801 pep4* pESC-LEU-**InsMCM2**-**InsMCM7** pESC-TRP-MCM6-**InsMCM4** pESC-URA-**InsHA_MCM3**-MCM5	2,3,4,7 Ins
YC246	*MATa bar1Δ leu2-3,112 ura3-52 his3-Δ200 trp1-Δ 63 ade2-1 lys2-801 pep4* pESC-LEU-**InsMCM2**-**InsMCM7** pESC-TRP-MCM6-MCM4 pESC-URA-**InsHA_MCM3**-**InsMCM5**	2,3,5,7 Ins
YC247	*MATa bar1Δ leu2-3,112 ura3-52 his3-Δ200 trp1-Δ 63 ade2-1 lys2-801 pep4* pESC-LEU-**InsMCM2**-**InsMCM7** pESC-TRP-MCM6-**InsMCM4** pESC-URA-HA_MCM3-**InsMCM5**	2,4,5,7 Ins
YC248	*MATa bar1Δ leu2-3,112 ura3-52 his3-Δ200 trp1-Δ 63 ade2-1 lys2-801 pep4* pESC-LEU-**InsMCM2**-**InsMCM7** pESC-TRP-**InsMCM6**-**InsMCM4** pESC-URA-HA_MCM3-MCM5	2,4,6,7 Ins
YC249	*MATa bar1Δ leu2-3,112 ura3-52 his3-Δ200 trp1-Δ 63 ade2-1 lys2-801 pep4* pESC-LEU-**InsMCM2**-**InsMCM7** pESC-TRP-**InsMCM6**-MCM4 pESC-URA-HA_MCM3-**InsMCM5**	2,5,6,7 Ins
YC250	*MATa bar1Δ leu2-3,112 ura3-52 his3-Δ200 trp1-Δ 63 ade2-1 lys2-801 pep4* pESC-LEU-**InsMCM2**-**InsMCM7** pESC-TRP-MCM6-**InsMCM4** pESC-URA-**InsHA_MCM3**-**InsMCM5**	2,3,4,5,7 Ins
YC251	*MATa bar1Δ leu2-3,112 ura3-52 his3-Δ200 trp1-Δ 63 ade2-1 lys2-801 pep4* pESC-LEU-**InsMCM2**-**InsMCM7** pESC-TRP-**InsMCM6**-**InsMCM4** pESC-URA-**InsHA_MCM3**-MCM5	2,3,4,6,7 Ins
YC255	*MATa bar1Δ leu2-3,112 ura3-52 his3-Δ200 trp1-Δ 63 ade2-1 lys2-801 pep4* pESC-LEU-**InsMCM2**-**InsMCM7** pESC-TRP-**InsMCM6**-MCM4 pESC-URA-**InsHA_MCM3**-**InsMCM5**	2,3,5,6,7 Ins
YC256	*MATa bar1Δ leu2-3,112 ura3-52 his3-Δ200 trp1-Δ 63 ade2-1 lys2-801 pep4* pESC-LEU-**InsMCM2**-**InsMCM7** pESC-TRP-**InsMCM6**-**InsMCM4** pESC-URA-HA_MCM3-**InsMCM5**	2,4,5,6,7 Ins
YC257	*MATa bar1Δ leu2-3,112 ura3-52 his3-Δ200 trp1-Δ 63 ade2-1 lys2-801 pep4* pESC-LEU-MCM2-**InsMCM7** pESC-TRP-**InsMCM6**-**InsMCM4** pESC-URA-**InsHA_MCM3**-**InsMCM5**	3,4,5,6,7 Ins
YC258	*MATa bar1Δ leu2-3,112 ura3-52 his3-Δ200 trp1-Δ 63 ade2-1 lys2-801 pep4* pESC-LEU-**InsMCM2**-**InsMCM7** pESC-TRP-**InsMCM6**-**InsMCM4** pESC-URA-**InsHA_MCM3**-**InsMCM5**	2,3,4,5,6,7 Ins
YC259	*MATa bar1Δ leu2-3,112 ura3-52 his3-Δ200 trp1-Δ 63 ade2-1 lys2-801 pep4* pESC-LEU-**InsMCM2**-**InsMCM7** pESC-TRP-**InsMCM6**-MCM4 pESC-URA-**InsHA_MCM3**-MCM5	2,3,6,7 Ins
YC260	*MATa bar1Δ leu2-3,112 ura3-52 his3-Δ200 trp1-Δ 63 ade2-1 lys2-801 pep4* pESC-LEU-MCM2-**InsMCM7** pESC-TRP-**InsMCM6**-**InsMCM4** pESC-URA-**InsHA_MCM3**-MCM5	3,4,6,7 Ins
YC278	*MATa bar1Δ leu2-3,112 ura3-52 his3-Δ200 trp1-Δ 63 ade2-1 lys2-801 pep4* pESC-LEU-MCM2-MCM7 pESC-TRP-MCM6-**InsMCM4** pESC-URA-HA_MCM3-MCM5	4 Ins
YC279	*MATa bar1Δ leu2-3,112 ura3-52 his3-Δ200 trp1-Δ 63 ade2-1 lys2-801 pep4* pESC-LEU-MCM2-**InsMCM7** pESC-TRP-MCM6-MCM4 pESC-URA-HA_MCM3-MCM5	7 Ins
YC280	*MATa bar1Δ leu2-3,112 ura3-52 his3-Δ200 trp1-Δ 63 ade2-1 lys2-801 pep4* pESC-LEU-**InsMCM2**-MCM7 pESC-TRP-MCM6-**InsMCM4** pESC-URA-HA_MCM3-MCM5	2,4 Ins
YC281	*MATa bar1Δ leu2-3,112 ura3-52 his3-Δ200 trp1-Δ 63 ade2-1 lys2-801 pep4* pESC-LEU-**InsMCM2**-**InsMCM7** pESC-TRP-MCM6-MCM4 pESC-URA-HA_MCM3-MCM5	2,7 Ins
YC282	*MATa bar1Δ leu2-3,112 ura3-52 his3-Δ200 trp1-Δ 63 ade2-1 lys2-801 pep4* pESC-LEU-MCM2-MCM7 pESC-TRP-MCM6-**InsMCM4** pESC-URA-**InsHA_MCM3**-MCM5	3,4 Ins
YC283	*MATa bar1Δ leu2-3,112 ura3-52 his3-Δ200 trp1-Δ 63 ade2-1 lys2-801 pep4* pESC-LEU-MCM2-**InsMCM7** pESC-TRP-MCM6-MCM4 pESC-URA-**InsHA_MCM3**-MCM5	3,7 Ins
YC284	*MATa bar1Δ leu2-3,112 ura3-52 his3-Δ200 trp1-Δ 63 ade2-1 lys2-801 pep4* pESC-LEU-MCM2-MCM7 pESC-TRP-MCM6-**InsMCM4** pESC-URA-HA_MCM3-**InsMCM5**	4,5 Ins
YC285	*MATa bar1Δ leu2-3,112 ura3-52 his3-Δ200 trp1-Δ 63 ade2-1 lys2-801 pep4* pESC-LEU-MCM2-MCM7 pESC-TRP-**InsMCM6**-**InsMCM4** pESC-URA-HA_MCM3-MCM5	4,6 Ins
YC286	*MATa bar1Δ leu2-3,112 ura3-52 his3-Δ200 trp1-Δ 63 ade2-1 lys2-801 pep4* pESC-LEU-MCM2-**InsMCM7** pESC-TRP-MCM6-**InsMCM4** pESC-URA-HA_MCM3-MCM5	4,7 Ins
YC287	*MATa bar1Δ leu2-3,112 ura3-52 his3-Δ200 trp1-Δ 63 ade2-1 lys2-801 pep4* pESC-LEU-MCM2-**InsMCM7** pESC-TRP-MCM6-MCM4 pESC-URA-HA_MCM3-**InsMCM5**	5,7 Ins
YC288	*MATa bar1Δ leu2-3,112 ura3-52 his3-Δ200 trp1-Δ 63 ade2-1 lys2-801 pep4* pESC-LEU-MCM2-**InsMCM7** pESC-TRP-**InsMCM6**-MCM4 pESC-URA-HA_MCM3-MCM5	6,7 Ins

Supplementary Table S3 (related to Experimental procedures): MCM2-7 plasmids used in this study

Plasmid number	plasmid name
pCS14	pESC-LEU-MCM2-MCM7
pCS15	pESC-TRP-MCM6-MCM4
pCS232	pESC-URA-HA_MCM3-MCM5
pCS265	pESC-LEU-InsMCM2-MCM7
pCS266	pESC-URA-InsHA_MCM3-MCM5
pCS267	pESC-TRP-InsMCM4-MCM6
pCS268	pESC-URA-HA_MCM3-InsMCM5
pCS269	pESC-TRP-MCM4-InsMCM6
pCS270	pESC-LEU-MCM2-InsMCM7
pCS271	pESC-URA-InsHA_MCM3-InsMCM5
pCS272	pESC-TRP-InsMCM4-InsMCM6
pCS278	pESC-LEU-InsMCM2-InsMCM7
pCS280	pESC-LEU-MCM2-MCM7_MBP-Mcm2

SUPPLEMENTARY REFERENCES

Evrin C, Clarke P, Zech J, Lurz R, Sun J, Uhle S, Li H, Stillman B, Speck C (2009) A double-hexameric MCM2-7 complex is loaded onto origin DNA during licensing of eukaryotic DNA replication. *Proc Natl Acad Sci U S A* **106:** 20240-20245

Evrin, C., Fernandez-Cid, A., Zech, J., Herrera, M.C., Riera, A., Clarke, P., Brill, S., Lurz, R. and Speck, C. (2013) In the absence of ATPase activity, pre-RC formation is blocked prior to MCM2-7 hexamer dimerization. Nucleic Acids Res, 41, 3162-3172.

Klemm RD, Austin RJ, Bell SP (1997) Coordinate binding of ATP and origin DNA regulates the ATPase activity of the origin recognition complex. *Cell* **88:** 493-502

Speck C, Chen Z, Li H, Stillman B (2005) ATPase-dependent cooperative binding of ORC and Cdc6 to origin DNA. *Nat Struct Mol Biol* **12:** 965-971

Speck C, Stillman B (2007) Cdc6 ATPase activity regulates ORC x Cdc6 stability and the selection of specific DNA sequences as origins of DNA replication. *J Biol Chem* **282:** 11705-11714
